# Novel Nine-Exon AR Transcripts (Exon 1/Exon 1b/Exons 2–8) in Normal and Cancerous Breast and Prostate Cells

**DOI:** 10.3390/ijms18010040

**Published:** 2016-12-27

**Authors:** Dong Gui Hu, Ross A. McKinnon, Julie-Ann Hulin, Peter I. Mackenzie, Robyn Meech

**Affiliations:** Department of Clinical Pharmacology and Flinders Centre for Innovation in Cancer, Flinders University School of Medicine, Flinders Medical Centre, Adelaide 5042, Australia; ross.mckinnon@flinders.edu.au (R.A.M.); julieann.hulin@flinders.edu.au (J.-A.H.); peter.mackenzie@flinders.edu.au (P.I.M.); robyn.meech@flinders.edu.au (R.M.)

**Keywords:** androgen receptor, alternative splicing, prostate cancer, breast cancer, PCGEM1

## Abstract

Nearly 20 different transcripts of the human androgen receptor (AR) are reported with two currently listed as Refseq isoforms in the NCBI database. Isoform 1 encodes wild-type AR (type 1 AR) and isoform 2 encodes the variant AR45 (type 2 AR). Both variants contain eight exons: they share common exons 2–8 but differ in exon 1 with the canonical exon 1 in isoform 1 and the variant exon 1b in isoform 2. Splicing of exon 1 or exon 1b is reported to be mutually exclusive. In this study, we identified a novel exon 1b (1b/TAG) that contains an additional TAG trinucleotide upstream of exon 1b. Moreover, we identified AR transcripts in both normal and cancerous breast and prostate cells that contained either exon 1b or 1b/TAG spliced between the canonical exon 1 and exon 2, generating nine-exon AR transcripts that we have named isoforms 3a and 3b. The proteins encoded by these new AR variants could regulate androgen-responsive reporters in breast and prostate cancer cells under androgen-depleted conditions. Analysis of type 3 AR-GFP fusion proteins showed partial nuclear localization in PC3 cells under androgen-depleted conditions, supporting androgen-independent activation of the AR. Type 3 AR proteins inhibited androgen-induced growth of LNCaP cells. Microarray analysis identified a small set of type 3a AR target genes in LNCaP cells, including genes known to modulate growth and proliferation of prostate cancer (*PCGEM1*, *PEG3*, *EPHA3*, and *EFNB2*) or other types of human cancers (*TOX3*, *ST8SIA4*, and *SLITRK3*), and genes that are diagnostic/prognostic biomarkers of prostate cancer (*GRINA3*, and *BCHE*).

## 1. Introduction

Androgen signalling through the androgen receptor (AR) is essential for human prostate growth and function; however, excessive AR-signalling activity in the prostate is well-known to be involved in prostate cancer development and progression [1]. Therefore, endocrine therapy, mainly through inhibition of AR-signalling activity by antiandrogens (e.g., flutamide, bicalutamide, etc.), has been the mainstay of hormone therapy for prostate cancer over the last few decades [2]. Although the majority of patients initially respond to antiandrogens, most treated patients develop castration-resistant prostate cancer (CRPC) after a median 18–36 months of antiandrogen treatment and eventually die from this disease [3]. The mechanisms that have long been known to contribute to the development of CRPC following endocrine therapy include AR amplification, AR mutation, altered expression of AR-coregulators, androgen-independent AR activation, and enhanced intratumoral androgen production [4].

The *AR* gene is located at Xq11-13 and contains eight canonical exons (exons 1–8) and at least seven “cryptic” exons (CE1–5, CE9, and exon 1b) [5,6,7]. There are only two androgen receptor transcript isoforms that are currently listed as NCBI reference sequences of the *AR* gene (RefSeq, assessed August 2016). Isoform 1 (NM_000044.3) consists of canonical exons 1–8 and encodes the full-length wild-type AR (NP_000035.2, termed type 1 AR in this study). Isoform 2 (NM_0010116445.2) comprises exon 1b spliced to exons 2–8 and encodes AR45 (NP_001011645.1, termed type 2 AR in the study), a variant AR protein that is highly expressed in the heart [8]. The six other known cryptic exons (CE1–5, CE9) appear in AR transcripts in prostate cancer cells via alterative splicing or due to genomic rearrangements within the *AR* gene, generating approximately 20 variant AR transcripts (e.g., AR-V1 to AR-V18, AR8, and AR23) [7,9,10,11,12,13]. Most of these ARVs are generated through splicing of exon 3 to a downstream cryptic exon, and thus encode variant AR proteins that contain the NTD (N-terminal transactivation domain encoded by exon 1) and DBD (DNA binding domain encoded by exons 2/3), followed by a C-terminal unique peptide of variable length [6,7]. These variant ARs lack the ligand-binding domain (LBD) encoded by exons 5–8; however, many of them have been shown to be constitutively (AR-V3, -V4, -V7, and -V12) [5,14,15,16] or conditionally (AR-V1 and AR-V9) [5,15] active androgen-independent transcription factors in prostate cancer cells. The constitutive androgen-independent activity of LBD-lacking AR variants is considered to be involved in the development of CRPC following endocrine therapy [5,6,10,15].

Breast cancers are highly heterogeneous diseases. Apocrine breast cancers, a subset of ER-negative and AR-positive breast cancers, show androgen-stimulated growth [17,18]. As such, phase I/II clinical trials of drugs (bicalutamide, enzalutamide, abiraterone) that repress androgen-mediated AR-signalling pathway have begun for treating this type of breast cancer and other advanced metastatic breast cancers (see clinicaltrials.gov: NCT00468715, NCT01597193, and NCT00755885). We recently reported the expression of AR variant transcripts and proteins (e.g., AR-V7) in breast cancer cell lines and breast cancer tissues, highlighting a potential role of AR variants in mediating resistance to antiandrogen therapy in breast cancer [9,19].

As mentioned earlier, both AR transcript isoforms 1 and 2 contain exons 2–8 but differ in the use of the canonical exon 1 or exon 1b. Splicing of exon 1 or exon 1b has been considered as mutually exclusive [8,20]. The present study identifies novel nine-exon AR transcripts that have exon 1b spliced between exon 1 and exon 2 in normal and cancerous breast and prostate cells. Expression studies demonstrate that the variant proteins encoded by these novel AR transcripts are biologically active androgen receptors, and microarray analysis indicates that they regulate several target genes that have known roles in prostate cancer.

## 2. Results

### 2.1. Identification of Transcripts with Exon 1b Spliced between Exon 1 and Exon 2 Suggesting the Existence of Novel Nine-Exon Androgen Receptor (AR) Variants

**Figure 1:**
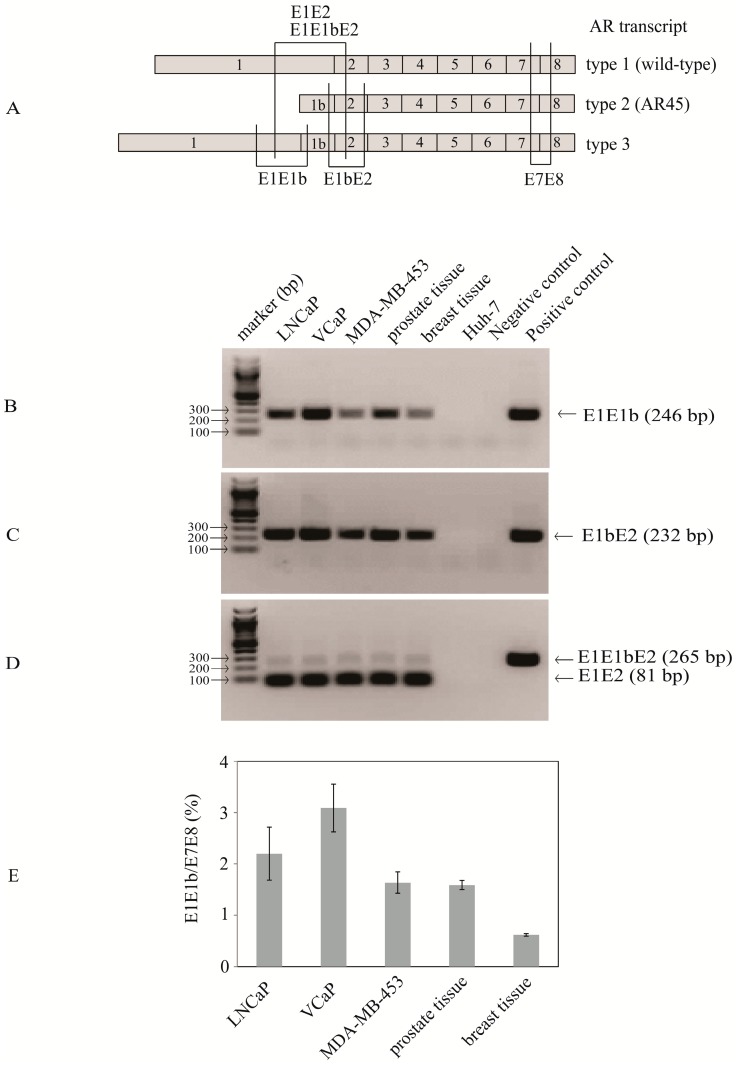
Expression of AR transcripts in breast and prostate cancer cell lines and normal breast and prostate cancer tissues. Primers were used to amplify the junction region of exon 1/exon 1b (E1E1b for type 3 AR only), exon 1b/exon 2 (E1bE2 for type 2 and type 3 AR), or exon 1/exon 1b/exon 2 (E1E2 for type 1 AR and E1E1bE2 for type 3 AR) using cDNA from normal breast and prostate tissues, MDA-MB-453, LNCaP, VCaP, and Huh-7 cells. Positive controls use pcDNA3/type 3a as template; negative controls use mock RT products (without RNA) as template. (**A**) Schematic showing regions of AR transcripts amplified via RT-PCR; (**B**–**D**) electrophoretic separation showing the expected sizes of RT-PCR products; and (**E**) quantitative RT-PCR analysis of type 3 AR (using E1E1b primers) and wild-type AR (using E7E8 primers). Expression levels of type 3 AR are shown as a percentage of wild-type AR (set to 100%). Data shown are means ± SEM of three independent experiments.

As mentioned above, AR isoforms 1 and 2 differ in that isoform 1 contains the classical exon 1 and isoform 2 contains the alternative exon 1b [8] (Figure 1). The splicing of exon 1 and exon 1b is considered to occur in a mutually exclusively manner [20]. To examine whether other variant forms of AR may exist, we amplified the AR from various breast and prostate cancer cell lines by RT-PCR using primers located in the canonical exon 1 and exon 8, and then cloned and sequenced a set of the PCR products. Among the sequenced clones we identified one (derived from cDNA of MDA-MB-453 cells) that contained exon 1 linked to exon 1b, followed by exons 2–8 (see Figure S1). This indicated that the splicing of exon 1 and exon 1b is not mutually exclusive and that novel nine exon AR transcripts (exon 1/exon 1b/exons 2–8) are expressed in breast cancer MDA-MB-453 cells. As the novel AR transcript contains both exon 1 of type 1 AR and exon 1b of type 2 AR, it is named herein type 3 AR.

Next, RT-PCR was used to examine the expression of the type 1, 2 and 3 AR transcripts in prostate cancer cell lines (VCaP, LNCaP) and normal breast and prostate tissues. Primers (Figure 1A) were designed that were specific for: (1) the junction of exon 1/exon 1b (E1E1b) that would detect type 3 AR only; (2) the junction of exon 1b/exon 2 (E1bE2) that would detect both type 2 and type 3 AR; and (3) the junction of exon 1/exon 2 that would detect both type 1 (E1E2) and type 3 (E1E1bE2) AR. RT-PCR analysis revealed the expression of all three types of AR transcripts in three AR-positive breast and prostate cancer cell lines (MDA-MB-453, VCaP, and LNCaP) and normal breast and prostate tissues but not in the AR-negative liver cancer Huh7 cell line (Figure 1B–D). Of note, the two bands shown in Figure 1D were amplified from the region of exon 1/exon 2 of type 1 AR transcript (an 81-bp product) and the region of exon 1/exon 1b/exon 2 of type 3 AR transcript (a 265-bp product).

Quantitative RT-PCR for the type 3 AR transcript (E1E1b) and for the type 1 AR transcript (E7/E8) indicated that type 3 AR was expressed at a level approximately 2%–3% of type 1 AR in breast and prostate tissues and cancer cell lines (Figure 1E). This level is similar to previous reports of other LBD-lacking AR variants (expressed at <5% of the levels of wild-type AR in breast and prostate cancer cell lines and tissues), although they may be expressed more highly in some prostate cancer cell lines and clinical prostate cancer specimens [5,15,21].

### 2.2. Type 3a and Type 3b AR Transcripts Are Defined by Two Different Exon 1bs

**Figure 2:**
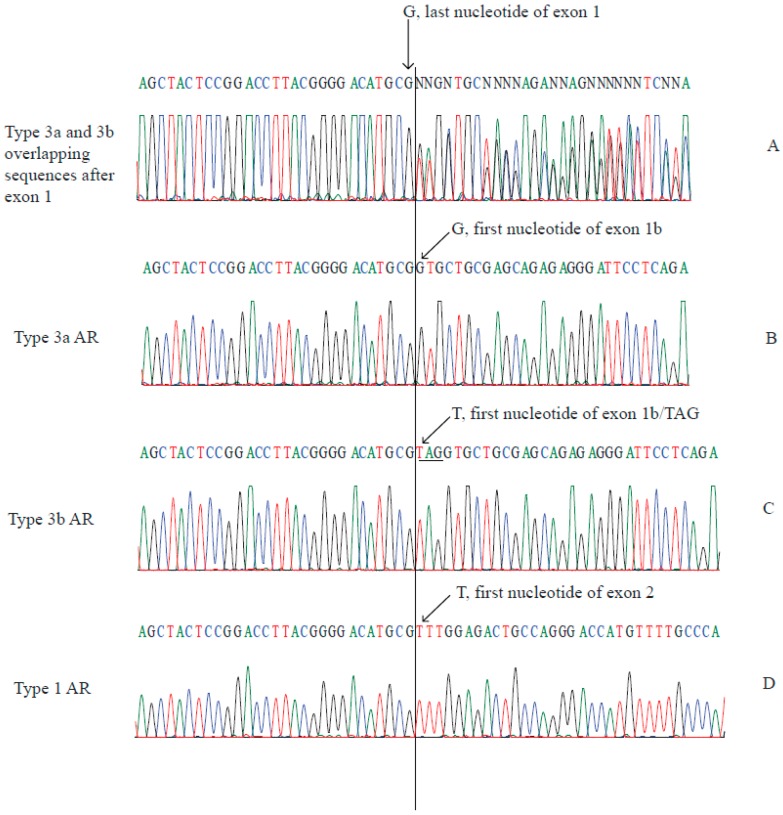
Chromatograms showing the sequences of RT-PCR amplicons spanning the exon 1/exon 1b region of type 3 AR transcripts (generated from VCaP cells): (**A**) direct sequencing of RT-PCR products showed two overlapping sequences after exon 1; (**B**,**C**) cloning of RT-PCR products followed by sequencing showed two different exon 1bs that generate type 3a (exon 1b) (**B**) and type 3b (exon 1b/TAG) (**C**) AR transcripts; and (**D**) sequence of the exon 1/exon 2 junction from wild-type AR transcripts (type 1 AR) for comparison. The nucleotides of A, C, G, T in chromatograms are in green, blue, black, and red, respectively. The curved line defines the junction between exon 1 and exon1b/exon1b/TAG (A), exon 1b (**B**), exon 1b/TAG (**C**), or exon 2 (**D**).

Sequencing of the RT-PCR products that were amplified using primers that span the exon 1/exon 1b junction (E1E1b) from three cell lines revealed two overlapping sequences following the last nucleotide G of exon 1 (Figure 2A). To define the two overlapping sequences, these RT-PCR products were cloned into a vector and 23 clones (9, 9, and 5 clones from MDA-MB-453, LNCaP, and VCaP, respectively) were sequenced. Six clones (2, 1, and 3 clones from MDA-MB-453, LNCaP, and VCaP, respectively) were shown to have the expected exon 1/exon 1b junction (Figure 2B); however, 17 clones (7, 8, and 2 clones MDA-MB-453, LNCaP, and VCaP, respectively) had an additional “TAG” trinucleotide (underlined) inserted between exon 1 and exon 1b (Figure 2C).

**Figure 3:**
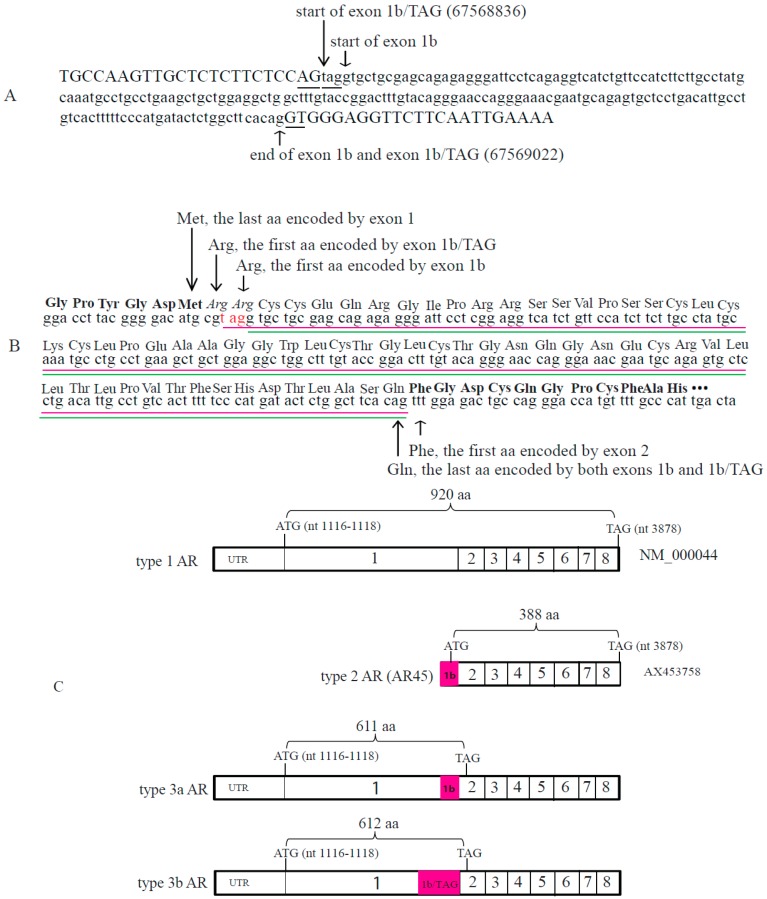
Genomic positions and sequences of exon 1b and exon 1b/TAG and the structures of three types of AR transcripts: (**A**) Positions and genomic sequences of exon 1b and exon 1b/TAG. Genomic coordinates are given according to GRCh38/HG38. Underlined are the dinucleotides AG and GT that position before and after exon 1b/TAG and the trinucleotide TAG of exon 1b/TAG that is upstream of exon 1b; (**B**) Predicted translation of type 3a and 3b AR variants: shown are the last six amino acids (bold) encoded by exon 1, followed 62 or 63 amino acids encoded by exon 1b (underlined in Green) or exon 1b/TAG (underlined in red), and 11-amino acids (bold) encoded by the first 33 nucleotides of exon 2. The amino acid Arginine (Arg) that is inserted because of the addition of the trinucleotide “tag” (in red) in type 3b AR and the neighbouring Arg that is present in type 3a AR are in italic; (**C**) The transcript structures of type 1, 2, 3a, and 3b ARs. Type 1, 3a, and 3b ARs have the same initiation ATG codon in exon 1 (nucleotides 1116-1118 according to AR reference sequence NM_000044). Type 1 AR transcript encodes wild-type AR protein (920 aa); Type 2 AR transcript (AX453758) uses an initiation ATG codon in exon 1b and encodes the AR45 protein (388 aa); Type 3a and type 3b AR transcripts encode type 3a (611 aa) and type 3b (612 aa) AR proteins, respectively. Of note, exon 1b and exon 2 are translated in a different frame in type 3a/b AR than in type 2 AR. Exon 1b or exon 1b/TAG is highlighted in red. UTR, untranslated region.

Interrogation of genomic sequence showed that the novel exon 1b (termed exon 1b/TAG) begins three nucleotides (TAG) upstream of exon 1b (Figure 3A). Both exon 1b and exon 1b/TAG have the same sequence at their 3′-end and the exonic region is flanked by dinucleotide “GT” (underlined). Moreover the 5′ ends of both exon 1b and exon 1b/TAG are flanked by the dinucleotide “AG” (underlined). These flanking dinucleotides comply with the “GT-AG” rule that defines the start and end of highly conserved introns. For simplicity, we have named the exon 1b- and exon 1b/TAG-containing type 3 AR transcripts as type 3a AR and type 3b AR, respectively.

Type 1 and types 3a and 3b transcripts have the same translation start site in the canonical exon 1 (ATG at nucleotides 1116-1118, NM_000044); however, the open reading frame of the type 3a (or 3b) transcript reads through exon 1b (1b/TAG) and 33 nucleotides into exon 2 before reaching a premature stop codon (Figure 3B). The diagram in Figure 3C shows the structures of type 1, 2, 3a and 3b AR transcripts and predicted proteins. Briefly, type 1 AR transcript (NM_000044) encodes the wild-type AR (920 aa), and type 2 AR transcript (AX453758) encodes AR45, a variant AR (388 aa), containing the C-terminal 381 aa of type 1 AR encoded by exons 2–8 and a novel N-terminal 7 aa-peptide encoded by exon 1b. The type 3a AR transcript encodes a novel 611 aa AR protein containing the N-terminal 538 aa of type 1 AR encoded by exon 1 and a novel C-terminal 73 aa-peptide encoded by exons 1b and 2 (translated in a different frame to that used in AR45). The addition of the “TAG” trinucleotide at the 5′-end of exon 1b/TAG compared to exon 1b inserts an additional Arginine residue such that type 3b AR transcript encodes a 612 aa AR protein. Both predicted type 3 AR proteins contain the NTD (encoded by exon 1) but lack the DBD (encoded by exons 2/3) and LBD (encoded by exons 5–8) regions. Type 3 AR transcripts have a stop codon in exon 2, whereas the wild-type (type 1) AR transcripts have a stop codon in exon 8. The lower levels of type 3 AR transcripts compared to type 1 AR transcripts may be partly due to the premature stop codon that may invoke the non-sense-mediated mRNA decay (NMD) pathway [22].

### 2.3. Type 3 AR Variants Stimulated AR-Responsive Reporters in Prostate and Breast Cancer Cells in an Androgen-Independent Manner

**Figure 4:**
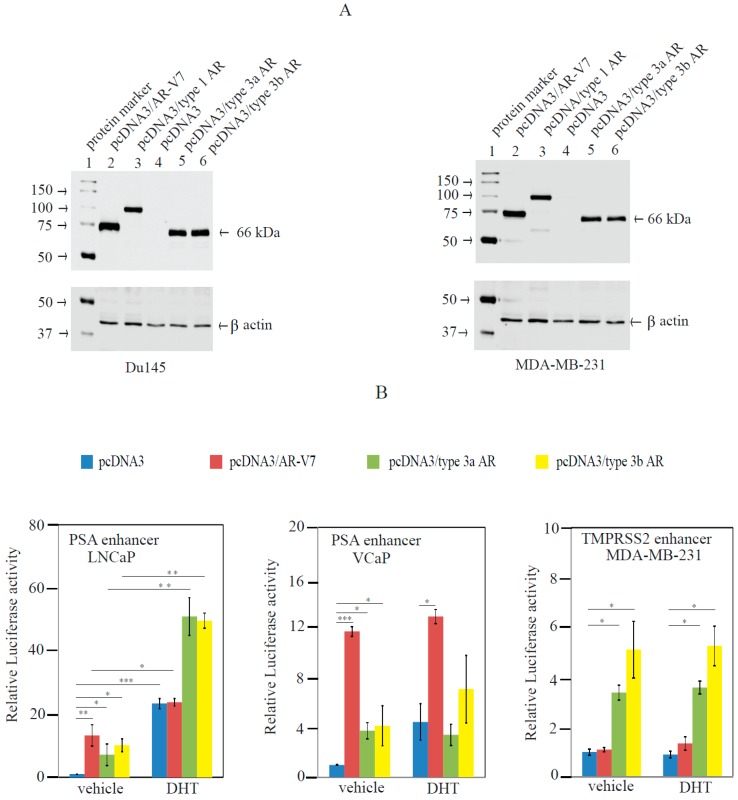
Type 3 ARs regulate AR-responsive reporters in prostate and breast cancer cells. (**A**) Western blot analysis was used to confirm expression of AR variants after transient transfection of relevant expression vectors (pcDNA3, pcDNA3/type 1 AR, pcDNA3/AR-V7, pcDNA3/type 3a AR, or pcDNA3/type 3b AR) in prostate cancer Du145 cells and breast cancer MDA-MB-231 cells. Proteins were detected using an anti-AR antibody (N20) and an anti-β-actin antibody as described in Materials and Methods; (**B**) Luciferase reporter co-transfection analyses were performed in VCaP, LNCaP, and MDA-MB-231 cells as described in the Methods. Briefly, an androgen-responsive reporter (pGL3/PSA enhancer or pGL3/TMPRSS2 enhancer) was co-transfected with an expression vector (pcDNA3, pcDNA3/AR-V7, pcDNA3/type 3a AR, or pcDNA3/type 3b AR) and cultured in stripped media/serum before treatment with 1 nM DHT or vehicle for 24 h. Luciferase activities were normalized as described in the Methods and reporter activity in cells transfected with the AR variants (with or without DHT) was expressed as a fold change over that in cells transfected with empty pcDNA3 vector and treated with ethanol (set to a value of 1). Data shown are means ± SEM of three independent experiments in triplicate. Statistical analyses used one-way analysis of variance (ANOVA) followed by Tukey’s *post hoc* multiple comparison test. *p* < 0.05 (*), *p* < 0.01 (**), *p* < 0.001 (***).

The full-length coding sequences of the type 3a and type 3b AR transcripts were cloned into the expression vector pcDNA3 generating “pcDNA3/type 3a AR” and “pcDNA3/type 3b AR” as described in Materials and Methods. The expression constructs were transfected into prostate cancer Du145 and breast cancer MDA-MB-231 cells. Expression constructs for wild-type AR (pcDNA3/type 1 AR) or the well-characterized AR-V7 variant (pcDNA3/AR-V7) [15] and empty vector (pcDNA3) were transfected as controls. Western blotting using an antibody (N-20) that recognizes the exon 1-encoded N-terminus of AR confirmed expression of both type 3a and type 3b AR proteins (approximately 66 kDa) (Figure 4A) as well as the wildtype and AR-V7 proteins. Attempts to develop a type 3 AR-specific antibody via immunization of rabbits using recombinant C-terminal novel 73 aa-peptide of type 3a AR variants was unsuccessful (data not shown). Hence, as with many other reported AR variants, it is not presently possible to accurately characterize the endogenous expression profile of type 3a and type 3b AR variant proteins. Despite this limitation, chemiluminescent Western blotting assays with very long exposure times were applied to assess whether the AR antibody (N20) could detect proteins with a molecular weight consistent with type 3 AR proteins in MDA-MB-231 and LNCaP cells. A 10-min exposure revealed such protein bands in MDA-MB-231 (see Figure S2A) and LNCaP cells (See Figure S2B), suggesting that type 3 AR proteins are endogenously expressed in these cells. Figure S2A also showed the expression of wild-type (type 1) AR protein at very low levels in MDA-MB-231 cells that were transfected with an empty pcDNA3 vector. This observation is consistent with previous reports of AR transcripts and proteins in this cell line [9,23]. Figure S2B showed that the expression of type 3 AR proteins was most evident in LNCaP cells that were cultured in media containing stripped serum. DHT treatment reduced type 3 AR protein levels and this was consistent with previous findings that androgens downregulated expression of multiple AR variants in breast and prostate cancer cell lines including LNCaP cells [9,21].

To determine the biological activity of type 3a and type 3b AR variants, co-transfection experiments were performed with luciferase reporters carrying known AR-responsive enhancers (pGL3/PSA enhancer or pGL3/TMPRSS2 enhancer) in prostate (LNCaP, VCaP) and breast (MDA-MB-231) cancer cell lines. The transactivation function of the type 3a and type 3b variants was compared to that of AR-V7, and transfected cells were treated with either 1 nM dihydrotestosterone (DHT) or vehicle to determine whether activity was ligand dependent or independent.

As expected based on previous reports, DHT alone stimulated the PSA enhancer reporter in AR-positive LNCaP and VCaP cells (Figure 4B, blue columns, left and middle graph), and did not affect the activity of the TMPRSS2 enhancer reporter in MDA-MB-231 cells (Figure 4B, blue columns, right graph). Moreover, consistent with previous reports, AR-V7 elevated the activity of the *PSA* enhancer reporter in LNCaP and VCaP cells in the absence of androgen (Figure 4B, red column, left and middle graph). AR-V7 was not able to enhance the activity of the TMPRSS2 enhancer reporter in MDA-MB-231 cells with or without DHT treatment (Figure 4B, red columns, right graph), although our recent evidence has shown that AR-V7 can stimulate the activity of a synthetic AR-responsive reporter in MDA-MB-453 and MFM-223 cells [19]. 

Both type 3a and type 3b AR variants stimulated the *PSA* enhancer reporter in LNCaP and VCaP cells in the absence of DHT (Figure 4B, green and yellow columns, left and middle graph), demonstrating their androgen-independent activation function, a hallmark of other LBD-lacking AR variants [7]. The stimulation of the PSA enhancer by type 3a and type 3b AR variants was unaffected by DHT in VCaP cells, but was enhanced by DHT in LNCaP cells. In breast cancer MDA-MB-231 cells, both type 3a AR and type 3b AR enhanced the activity of the TMPRSS enhancer reporter in the absence of androgen, and this activation was not affected by DHT treatment (Figure 4B, green and yellow columns, right graph).

We further examined the activity of type 3 AR variants in prostate cancer Du145 and PC3 cells and breast cancer MDA-MB-453 and MFM-223 cells but found no androgen-independent activation of the PSA or TMPRSS2 enhancer reporter in these cell contexts (data not shown). Collectively, these data reveal cell context-specific androgen-independent activation of AR-responsive reporters by type 3a and type 3b AR in prostate and breast cancer cells.

### 2.4. Type 3 AR Proteins Localize to Both Cytoplasm and Nuclei of PC3 Cells in Androgen-Depleted Conditions

**Figure 5:**
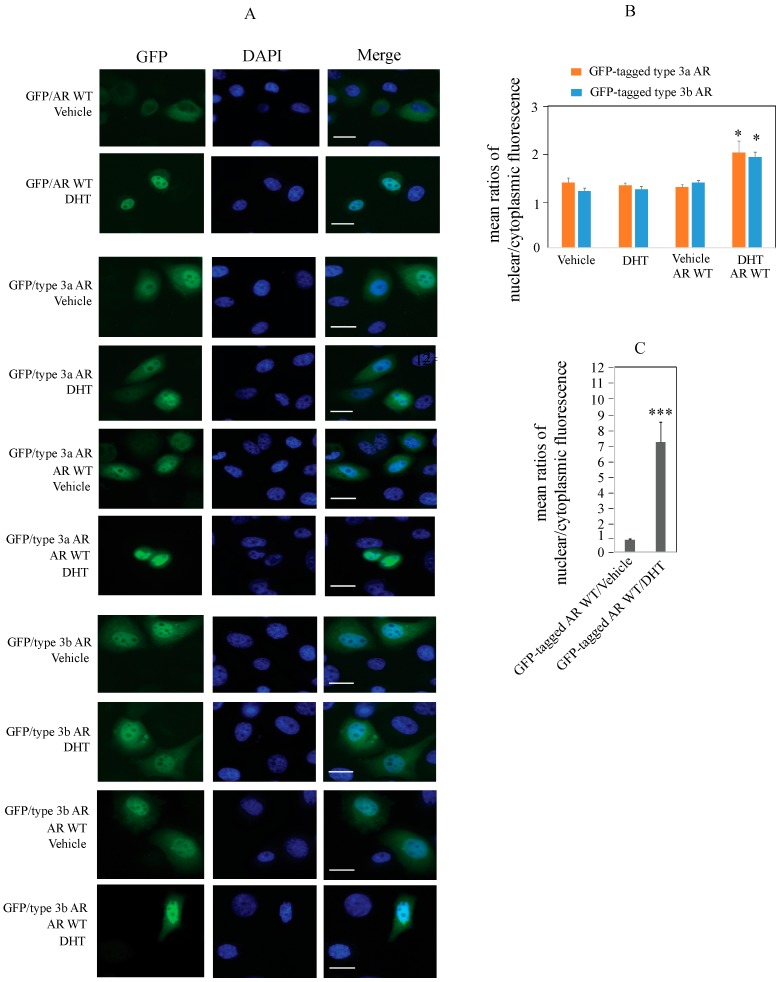
GFP-tagged type 3 AR proteins localize to both the cytoplasm and nuclei of AR-negative prostate cancer PC3 cells under androgen-depleted conditions; co-expression with wild-type AR enhances the nuclear localization of type 3 AR in the presence of DHT. (**A**) Fluorescence images of PC3 cells transfected with GFP-tagged wild-type AR (GFP/AR WT), GFP/type 3a AR, GFP/type 3b AR, or co-transfection of untagged AR WT with GFP-tagged type 3a or 3b AR (green) and DAPI (blue) after 4 h treatment with vehicle or 10 nM DHT. Scale bars represent 20 µm; (**B**) Average mean fluorescence ratio of nuclear:cytoplasmic GFP/type 3a AR or GFP/type 3b AR in vehicle- or DHT-treated PC3 cells; (**C**) Average mean fluorescence ratio of nuclear:cytoplasmic GFP/AR WT in vehicle- or DHT-treated PC3 cells. Fluorescence from a total number of 20–35 cells per condition was quantified as described under Methods. Error bars represent SEM. Statistical analysis was carried out by GraphPad Prism 6 (GraphPad Software, La Jolla, CA, USA) using two-way ANOVA (**B**) or independent *t*-test (**C**). *p* < 0.05 (*), *p* < 0.001 (***).

Although type 3 AR variants lack the DBD (encoded by exons 2–3) and the bipartite nuclear localization signal (NLS) (encoded by exons 3–4), we showed their androgen-independent activation of androgen-responsive reporters. This observation prompted us to define the cellular localization of type 3 AR proteins by expression of GFP/type 3 AR fusion proteins. To avoid potential interference from the endogenously expressed wild-type AR, AR-negative PC3 cells were used for these experiments. As shown in Figure 5, GFP/type 3a AR and GFP/3b type AR fusion proteins were localized to both cytoplasm and nuclei of PC3 cells in the absence of DHT (Figure 5A). The GFP fluorescence appeared greater in nuclei compared to cytoplasm (Figure 5A). This cytoplasmic/nuclear localization pattern was not significantly affected by DHT treatment, suggesting that this is a constitutive expression pattern (Figure 5B). By contrast, wild-type AR fused to GFP (GFP/AR WT) was mainly localized in the cytoplasm of PC3 cells (Figure 5A) in the absence of DHT but entered into the nuclei in the presence of DHT (Figure 5A,C). We co-expressed untagged wild-type AR with either GFP/type 3a AR or GFP/type 3b AR fusion proteins to assess whether the localization of type 3 AR was altered. The GFP/type 3a AR or GFP/type 3b AR fusion proteins showed enhanced nuclear localization when co-expressed with wild-type AR only in the presence of DHT (Figure 5A,B). The observation that wild-type AR facilitates the entry of type 3 AR proteins into nuclei of PC3 cells suggests that wild-type AR and type 3 AR proteins may interact.

### 2.5. Type 3 AR Variants Inhibited Androgen-Stimulated Growth of LNCaP Cells

**Figure 6:**
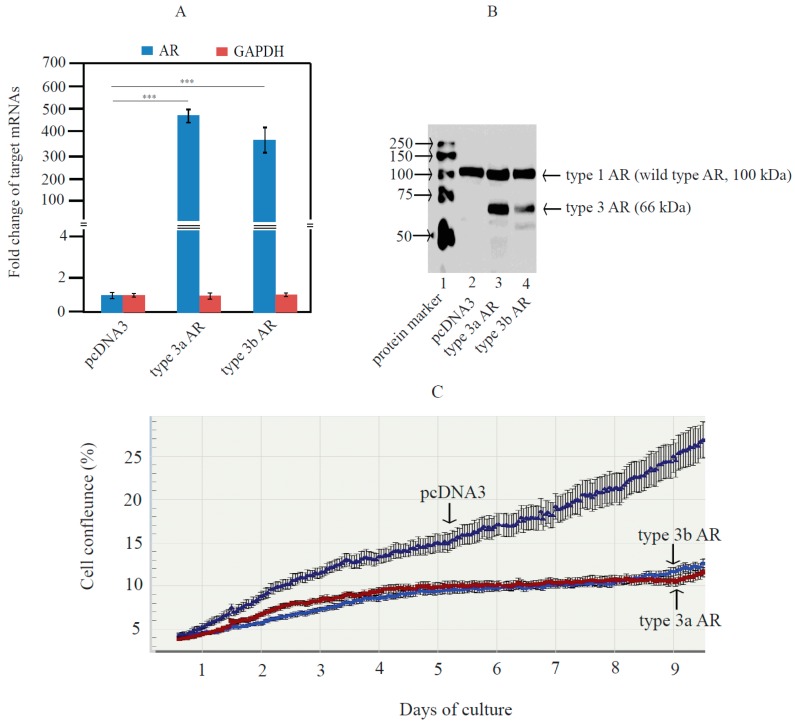
Type 3a and type 3b AR variants inhibit androgen-induced growth of LNCaP cells. (**A**) mRNA levels of type 3a or 3b AR as measured by qRT-PCR in control LNCaP cells and LNCaP cells stably expressing type 3a or 3b AR; Data shown are means ± SEM of fold changes of variant transcript levels in LNCaP cells stably expressing type 3a or 3b AR compared to the variant transcript levels in control LNCaP cells expressing no ectopic proteins from three independent experiments in triplicate. Statistical analysis was conducted by SPSS program using *t* test. *p* < 0.01 (***); (**B**) Expression of AR proteins in control LNCaP cells and LNCaP cells stably expressing type 3a or 3b AR as shown by Western blotting; (**C**) Growth of LNCaP cells that stably express type 3a AR or type 3b AR relative to control cells in the presence of DHT. Cell confluence (*y*-axis) was measured every 2 h over nine days of culture using the IncuCyte, and plotted as a function of time (*x*-axis).

The potential effect of type 3 AR variants on prostate cancer cell growth was assessed using LNCaP cells with stable heterologous expression of type 3a AR or type 3b AR. Heterologous expression of the AR variants in these cell lines was confirmed at both mRNA levels by RT-qPCR (Figure 6A) and protein levels by Western blotting assays (Figure 6B). The rate of cell growth with DHT stimulation was assessed over nine days using the IncuCyte real-time imaging system. Overexpression of type 3a AR or type 3b AR significantly inhibited androgen-induced LNCaP cell growth (Figure 6C) as compared to control LNCaP cells which were stably transfected with an empty pcDNA3 vector.

### 2.6. A Unique Set of Genes Regulated by Type 3a AR in LNCaP Cells as Revealed by Whole Genome Microarray Expression Profiling Assays

Whole-genome gene expression profiling was conducted using Affymetrix Gene Arrays to compare gene expression levels between LNCaP cells stably expressing type 3a AR and control LNCaP cells, with and without DHT treatment. As shown in Table 1, 10 genes including 2 miRNAs (*PCGEM1*, *BCHE*, *EFNB2*, *PSMD5*, *MBNL2*, *GSTA1*, *TLL1*, and *ZNF608*, has-miR-3689a, and has-miR-3689b) were down-regulated at similar levels by type 3a AR independent of DHT treatment. These results suggest that type 3a AR represses these genes in an androgen-independent manner. *PEG3* was the only gene that was upregulated by type 3a AR both with and without DHT treatment. Three genes (*SYT4*, *ST8SIA4*, and *LRRTM3*) were downregulated by type 3a AR only in the absence of DHT, and six genes (*COL12A*, *GRIN3A*, *SLITRK3*, *FAM198B*, *LRRN1*, and *TOX3*) were downregulated by type 3a AR only in the presence of DHT. One gene, *EPHA3*, was upregulated by type 3a AR only in the presence of DHT. Collectively, overexpression of type 3a AR altered the expression of 21 genes including two microRNAs in an androgen-dependent and/or -independent manner in LNCaP cells.

**Figure 7:**
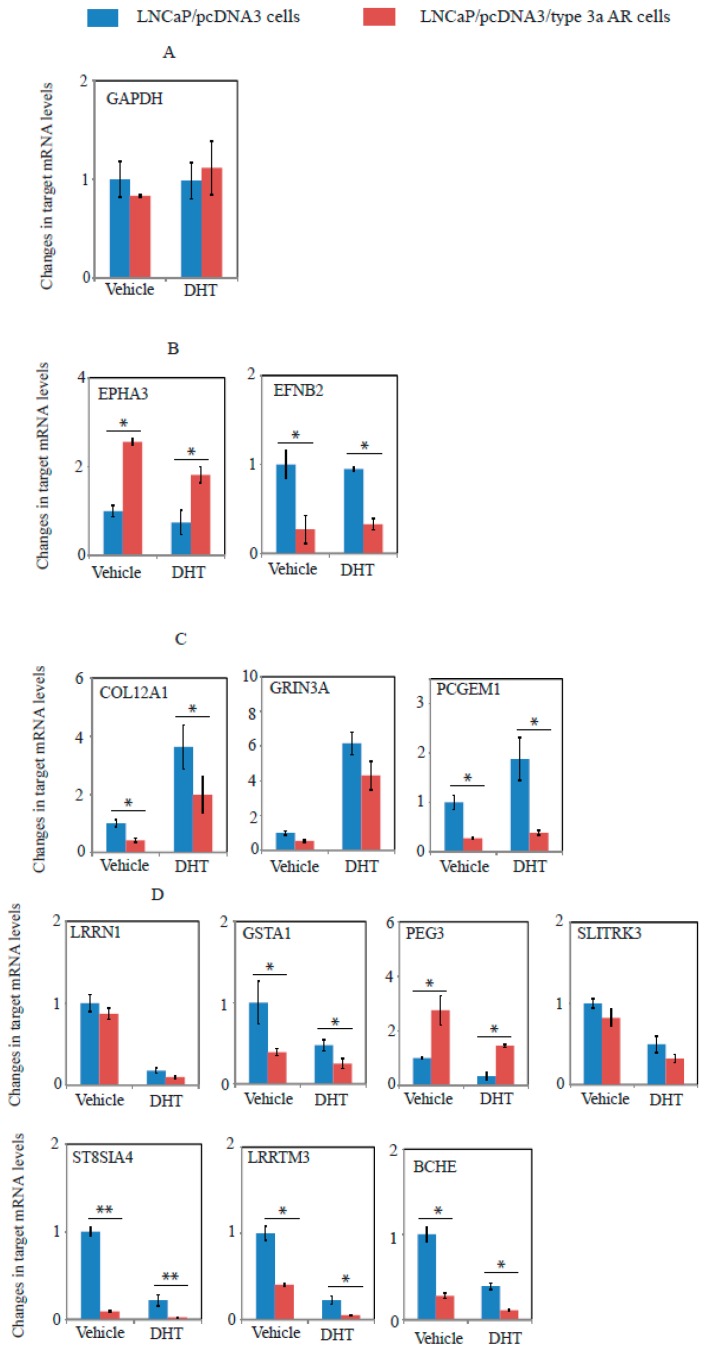
The expression of selected type 3a AR target genes (revealed by microarray analysis) is altered in LNCaP cells stably expressing type 3a AR (LNCaP/pcDNA3/type 3a AR) relative to control LNCaP cells (LNCaP/pcDNA3). Cells were cultured in stripped media/serum and treated with 10 nM DHT or vehicle for 24 h as described in the Methods. The expression level of each target gene in LNCaP/pcDNA3/type 3a AR cells with or without DHT is shown as the fold change relative to control LNCaP/pcDNA3 cells treated with vehicle (set to a value of 1). Shown are genes that are not affected by DHT (**A** and **B**), upregulated by DHT (**C**), and downregulated by DHT (**D**). Data shown are means ± SEM of three independent experiments. Statistical analysis used the SPSS program to perform *t* tests, *p* < 0.05 (*), *p* < 0.01 (**).

The expression levels of 12 of the genes identified as type 3a AR targets by Gene Array analysis were further measured by quantitative real-time RT-PCR (qRT-PCR) using gene-specific primers (Table S1). As shown in Figure 7, qRT-PCR results showed downregulation or upregulation of all 12 genes by type 3a AR, thus verifying the array data. Furthermore, 10 of these 12 genes were either upregulated (*COL12A1*, *GRIN3A*, and *PCGEM1*) (Figure 7C) or down-regulated (*LRRN1*, *GSTA1*, *PEG3*, *SLITRK3*, *BCHE*, *ST8SIA4*, and *LRRTM3*) (Figure 7D) by DHT in LNCaP cells. Androgen-mediated upregulation of *PCGEM1* [24,25] and downregulation of *LRRN1* [26] and *PEG3* [27] in LNCaP cells were previously reported. Type 3a AR repressed the expression of seven genes (*COL12A1*, *GRIN3A*, *PCGEM1*, *GSTA1*, *BCHE*, *ST8SIA4*, and *LRRTM3*) in the absence of DHT (Figure 7), and further decreased the expression of four of them (*GSTA1*, *BCHE*, *ST8SIA4*, and *LRRTM3*) following DHT stimulation. These results indicate that type 3a AR represses the expression of these androgen-downregulated genes under androgen-depleted condition.

## 3. Discussion

Transcripts corresponding to AR isoform 1 and 2 contain exon 1 and exon 1b, respectively, spliced upstream of exons 2–8. It has been suggested that the splicing of exons 1 and 1b might be mutually exclusive events [8,20]. The present study identified a novel form of exon 1b (1b/TAG) that includes the trinucleotide TAG immediately upstream of the previously described exon 1b sequence. Moreover, novel nine-exon transcripts that contain exon 1b (or exon 1b/TAG) spliced between exon 1 and exon 2 followed by exons 3–8, were found in normal and cancerous breast and prostate cells. These variant transcripts, termed isoforms 3a and 3b, contain an open reading frame that starts with the canonical AR initiation codon and end at a stop codon located at nucleotides 34–36 of exon 2. Thus the predicted type 3a and 3b protein products contain the NTD followed by a novel 73 aa or 74 aa C-terminal peptide, and lack both the DBD and LBD.

We showed that GFP-tagged type 3a and 3b AR proteins were present in both cytoplasm and nuclei of PC3 cells under androgen-depleted conditions and that this cytoplasmic/nuclear localization pattern was not significantly affected by DHT. The latter result is consistent with the lack of the LBD in the variants. Nuclear localization signals (NLS) are short basic amino acid-rich (lysine/arginine, K/R) peptide motifs involved in the nuclear import of proteins (>45 kDa) via their binding to importins through the nuclear pore complexes [28,29]. Classical NLSs may be monopartite NLS and bipartite NLS. Monopartite NLSs have a single K/R-rich motif (K(K/R)X(K/R)) whereas bipartite NLSs have two K/R-rich motifs that are separated by 10–12 aa linker ((K/R)(K/R)X_10–12_ (K/R)_3/5_) [28]. The wild-type AR has a bipartite NLS (^617^617RKCYEAGMTLGARKLKK^633^) that is essential for androgen-induced nuclear localization [30]. The first (RK) and second (RKLKK) segments of this bipartite NLS are encoded by exon 3 and exon 4, respectively. The nuclear localization of type 3a and 3b AR was unexpected as they both lack the conventional AR bipartite NLS. Studies with other AR variants lacking the second segment of the AR bipartite NLS showed either exclusive nuclear localization (AR-V7) or exclusive cytoplasmic localization (AR-V1 and AR-V9) [5,21,31]. The novel peptide encoded by the cryptic exon CE3 in AR-V7 contains a functional non-classical monopartite NLS (KFRV) at the equivalent position of the second segment of the wild-type AR bipartite NLS that is believed to be able to reconstruct the bipartite NLS for AR-V7 nuclear localization [5,21]. Both type 3a and 3b AR proteins have a putative non-classical monopartite NLS (RGIPRR) at the N-terminal end of the novel peptide encoded by exon 1b/1bTAG (Figure 3B). A similar non-classical monopartite NLS (KRAAER) in *BRCA1* has recently been shown to mediate nuclear localization of proteins encoded by *BRCA1* splice variants lacking the classical NLS [32]. The presence of type 3a and 3b AR in the cytoplasm might be related to a reduced efficiency of this non-classical NLS compared to classical NLSs. Further studies are required to verify the functionality of the putative NLS of type 3 AR.

Previously described AR variants that lack the LBD, such as AR-V7, contain the DBD that is involved in binding to target genes and androgen-independent activation [6,7,10]. Despite lacking the DBD, type 3a and 3b AR demonstrated androgen-independent activation of androgen-responsive reporters, and also altered the expression of known AR-target genes in prostate and breast cancer cells, suggesting that the variant AR proteins interact with DNA. It is possible that this interaction is mediated in part by dimerization with wildtype AR. Recent work showed that heterodimerization of wildtype AR with truncated ARs such as AR-V7 involves both the N-terminal FxxLF motif and the D-box located in the DBD; however these two motifs are redundant and loss of either one does not abrogate dimerization [33]. We showed that co-expression of untagged wild-type AR with GFP-tagged type 3a or 3b AR significantly enhanced nuclear localization of GFP/type 3 AR fusion proteins following DHT stimulation, suggesting a possible heterodimerization between wild-type AR and type 3 AR through the NTD; however, further studies are required to verify this possibility.

The functional significance of type 3 AR variant proteins may depend on the level of their endogenous expression. Western blotting assays with the AR antibody (N20) using long exposure times revealed low levels of endogenous proteins with the same molecular weight as heterologously expressed type 3 AR proteins in LNCaP and MDA-MB-231 cells. However, the endogenous expression levels of most AR variant proteins, including AR45, remain poorly defined due to the lack of such variant-specific antibodies [8,20], and their functions have mainly been defined by overexpression studies, such as those described here. Overexpression of LBD-lacking AR variants (e.g., AR-V7, and AR^v567es^) has been shown to promote prostate cancer growth both in cell lines and in mouse xenograft models under androgen-depleted conditions [5,14,15,16,21], presumably due to their androgen-independent activation of the AR signalling pathway. By contrast, the present study showed that overexpression of type 3a or type 3b AR inhibited androgen-dependent growth of LNCaP cells. Several early studies with androgen-insensitive prostate cancer PC3 cells showed that overexpression of wild-type AR inhibited cell growth [34,35]. More recent studies demonstrated that overexpression (2–6-fold) of wild-type AR proteins did not affect LNCaP cell growth when treated with 10 nM DHT and stimulated cell growth at 1 nM DHT [36,37]. Chan et al recently showed that overexpression of AR variant proteins (e.g., AR-V7, and AR^v567es^) at low levels stimulated LNCaP cell growth, but at higher levels (higher than endogenous wild-type AR protein) the variants inhibited cell growth [38]. We showed inhibition of LNCaP cell growth by type 3 AR proteins at a level that was equivalent (type 3a AR) or lower (type 3b AR) than that of endogenously expressed wild-type AR proteins (Figure 6B). We propose that this inhibition of LNCaP cell growth by type 3 AR may be related to altered expression of particular target genes as shown by array analysis, a subset of which will be discussed here.

Twelve of the genes regulated by type 3a AR in prostate cancer LNCaP cells (Table 1) have been previously linked to prostate cancer, including *PCGEM1* [24], *BCHE* [39,40], *PEG3* [27,41], *GSTA1* [42], *GRIN3A* [43,44], *LRRN1* [26,39], *MBNL2* [45], *EFNB2* [46], *SLITRK3* [1,47], *EPHA3* [48], *TOX3* [49], and *COL12A1* [50]. Four of these genes (*PCGME1*, *PEG3*, *EPHA3*, and *EFNB2*) are of particular interest as they are known to modulate cancer cell proliferation and/or survival.

Prostate cancer gene expression marker 1 (*PCGEM1*) encodes a prostate-specific long non-coding RNA (lncRNA) that has oncogenic activity in prostate cancer [24,51]; polymorphisms in *PCGEM1* have been linked to prostate cancer risk [52]. In LNCaP cells, overexpression of *PCGEM1* stimulated cell growth while knockdown of *PCGEM1* inhibited growth [24,53]. Downregulation of PCGEM1 by type 3 AR in the present study is thus consistent with the ability of type 3 AR to inhibit LNCaP cell growth. PCGEM1 participates in multiple pathways that impact on cancer growth including repression of the tumour suppressor miR-145 [54]. PCGEM1 is reported to delay induction of p53 and its downstream target p21 and inhibit cleavage of caspase 7 and PARP upon exposure of cancer cells to anthracyclines [51,53]. Intriguingly, PCGEM1 is also an AR co-activator that interacts with wild-type AR or its variants (e.g., AR-V7) to enhance ligand-dependent and ligand-independent AR-mediated gene activation programs and proliferation [55]. A very recent study has shown the splicing of AR-V7 in prostate cancer cells is controlled by PCGEM1 via its interaction with splicing factors hnRNP A1 and U2AF65 [56]. Interaction of PCGEM1 with hnRNP A1 suppresses AR-V7 synthesis via exon skipping whereas interaction of PCGEM1 with U2AF65 promotes AR-V7 synthesis via exonization. The observation that type 3a AR downregulates *PCGEM1* suggests the possibility that AR variants can modulate each other’s biogenesis. It is also possible that PCGEM1 influences splicing of type 3 AR, and this remains to be investigated. *PEG3* (paternally expressed gene 3) has a critical role in apoptosis, promoting the p53-mediated cell death pathway [57], and also inducing apoptosis via the tumour necrosis factor (TNF)/NFκB signal transduction pathway [58]. Dysregulation of the EPH receptor tyrosine kinase/ephrin signalling is implicated in cancer progression [59]. EPHA3 (EPH receptor A3) is upregulated in a variety of human cancers including prostate cancer [48], and stimulates prostate cancer proliferation and survival in LNCaP cells and mouse xenograft models [48]. EFNB2 (ephrin-B2) is a primary ligand of EPH receptor B4 (EPHB4). Downregulation of EFNB2 in CRPC compared to hormone-naïve prostate cancer was reported [60]. In prostate cancer 22Rv1 and breast cancer MCF7-10A cells, EPHB4 suppressed cancer cell growth via EPHB4/EFB2 signalling pathway in the presence of EFNB2; however, EPHB4 promoted cancer cell growth in the absence of EFNB2 [46]. Therefore, the dual function of EPHB4 as tumour promoter and suppressor is controlled by the absence and presence of EFNB2 in cancer cells. EFNB2 was also reported to drive perivascular invasion and proliferation of glioblastoma stem-like cells [61].

Three type 3a AR-regulated genes are reported to be useful diagnostic or prognostic biomarkers of prostate cancer, although their molecular mechanisms remain unclear. Bianchi-Frias et al recently reported over-expression of *GRIN3A* and *LRRN1* in urine specimens of high-grade prostate cancer and a 4-gene panel (*RELN*, *GRIN3A*, *RGS5* and *LRRN1*) that discriminated clinically relevant high-grade (Gleason score ≥ 4 + 3) tumours from low-grade (Gleason score ≥ 3 + 4) tumours using urine sediments [39]. Advanced prostate cancer is associated with a decreased activity of butyrycholinesterase (BCHE) [62] and BCHE is downregulated in androgen-independent prostate cancer C4-2 cells as compared to androgen-dependent LNCaP cells [63]. Multivariate analysis revealed that BCHE was significantly associated with biochemical recurrence-free survival (BRFS) in prostate cancer patients treated with radical prostatectomy [40].

In addition to the seven genes discussed above, other type 3a AR-regulated genes are linked to prostate cancer or other types of cancer. Glutathione *S*-transferases (GSTs) are involved in detoxification of a variety of carcinogens and thus prevent carcinogenesis [64]. Low levels of GSTA1 and GSTP1 are suggested to contribute to prostate carcinogenesis [42,65,66,67]. SLITRK3, a member of SLIT and neurotropic tyrosine receptor kinase (NTRK) like family (SLITRKs), was upregulated in brain tumour [68], lymphoma [69], and gastrointestinal stromal tumour (GIST) [70], and was one of three genes (*RTN4*, *SLITRK3*, and *SPON2*) found to define a cell differentiation signature for LNCaP cells [47]. ST8SIA4 (ST8 α-*N*-Acetyl-Neuraminide α-2,8-Sialyltransferase 4) synthesizes polysialic acid that is present on the embryonic neural cell adhesion molecule (N-CAM). siRNA knockdown of N-CAM and ST8SIA4 abolished pancreatic cancer cell aggregation and migration [71]. A missense mutation of ST8SIA4 (R168S) was reported as one of the driver mutations in glioblastoma [72]. The *TOX3* gene encoding a TOX (thymocyte selection-associated high mobility group box)-family transcription factor [73], resides in a genomic region (16q12) that is commonly deleted in breast cancers and is associated with breast cancer risk [73,74,75,76,77,78,79] although paradoxical functional studies suggest that it likely to have context specific functions in different forms of breast cancer [80]. The roles, if any, of the genes discussed here in controlling prostate cancer cell growth downstream of type 3 AR expression remain to be determined.

## 4. Materials and Methods

### 4.1. Total RNA of Normal Breast and Prostate Tissues

Total RNA of normal breast tissue was purchased from Clontech (Scientifix Pty Ltd., Mitcham VIC, Australia). Total RNA of normal prostate tissue was purchased from Ambion™ (Life Technologies Australia Pty Ltd., Scoresby, VIC, Australia).

### 4.2. Cell Culture, RNA Extraction, and Reverse Transcription

Prostate (VCaP, LNCaP) and breast (MDA-MB-453, MDA-MB-231) cancer cell lines were purchased from American Type Culture Collection (ATCC). Cell lines were maintained in either Dulbecco’s Modified Eagle Medium (DMEM) supplemented with 10% foetal bovine serum (FBS) (VCaP) or Roswell Park Memorial Institute (RPMI) 1640 Medium containing 5% FBS (LNCaP, MDA-MB-231, MDA-MB-453). For RNA extraction, cells were cultured in 6-well plates to 90% confluence and then harvested for RNA extraction using RNeasy Mini Kits (QIAGEN, Valencia, CA, USA) according to the manufacturer’s instructions. Reverse transcription (RT) was conducted as previously reported using reagents from Invitrogen [81]. Briefly, 1 µg of total RNA was incubated with DNase I at room temperature for 15 min and then subjected to reverse transcription at 50 °C for 50 min in 20 µL of first strand synthesis buffer (50 mM Tris-HCl, pH 8.0, 75 mM KCl, and 3 mM MgCl_2_) containing 50 units of superscript^®^ III reverse transcriptase, random hexamer primers, dNTPs, 50 units of RNaseOut recombinant ribonuclease inhibitor. The resultant cDNA was diluted five times with water, and 3 µL of each diluted cDNA sample (~60 ng) was used as template for each standard polymerase chain reaction (PCR).

### 4.3. Cloning of RT-PCR Products Corresponding to AR Variants

Primers (forward primer: 5′-CTTAGGATCC*ATG*GAAGTGCAGTTAGGGCT-3′ and reverse primer: 5′-AAATGCGGCCGC*TCA*CTGGGTGTGGAAATAG-3′) were designed to amplify the full-length coding region of the *AR* gene from cDNA samples of VCaP, LNCaP, or MDA-MB-453 cells. The initiation (*ATG*) and stop (*TCA*) codons of the *AR* gene in the forward and reverse primers respectively are in italics and BamHI and XhoI sites (underlined) used for cloning in the forward and reverse primers respectively are underlined. PCR was conducted by an iCycler (Bio-Rad, San Diego, CA, USA) in 50 µL of 1× Phusion GC buffer containing 1 unit of Phusion^®^ High-Fidelity DNA polymerase (Thermo Scientific, Scoresby, VIC, Australia) and 100 ng of cDNA from VCaP, LNCaP, or MDA-MB-453 cells. Resultant PCR products were then cloned into the TA vector (pCR^®^2.1, Invitrogen, Scoresby, VIC, Australia). Inserts were sequenced bidirectionally using primers T7 and Sp6 by the Flinders Sequencing Facility (Adelaide, SA, Australia).

### 4.4. Quantitative RT-PCR

Quantitative RT-PCR (qRT-PCR) was performed using a RotorGene 3000 instrument (Corbett Research, Mortlake, VIC, Australia) in a 20-µL reaction of 1xQuantiTect SYBR Green PCR master mix (QIAGEN) containing ~60 ng of cDNA sample and target gene-specific primers (Table S1). The mRNA levels of target genes relative to 18S rRNA were quantified using the 2^−ΔΔ*C*t^ method [82]. Quantification of wild-type (type 1 AR) and type 3 AR transcripts using real-time PCR was conducted essentially as reported in [9]. The primers amplifying the exon 7/exon 8 junction for measuring the levels of wild-type AR transcript were the same as reported in [9]. For quantifying type 3 AR transcripts, primers (forward primer: 5′-CACTTGTGTCAAAAGCGAAATG-3′ and reverse primer: 5′-TACAAAGTCCGGTACAAAGC-3′) were designed to amplify the exon 1/exon 1b region of type 3 AR transcripts. The resultant amplicon was cloned into the PCR-blunt vector (Invitrogen) according to manufacturer’s instructions to generate standard calibration curves for absolute quantification. For this purpose, four serial 10-fold dilutions containing known copy numbers (e.g., 18,000, 1800, 180, and 18) of target-containing plasmids were included in each qPCR run in order to calculate copy numbers of target transcripts in the experimental samples being amplified in the same run. The amplification conditions consisted of an initial activation step of 95 °C for 15 min, and 40 cycles of 95 °C for 10 s, 58 °C for 15 s, and 72 °C for 20 s. Data were obtained during the 72 °C extension phase of each cycle and analysed by RotorGene 6.1 (Corbett Research). Copy numbers of 18S rRNA transcripts were used to normalize the amount of total RNA amplified in each reaction. Each sample was amplified in duplicate and the resultant mean values were used for analysis. The expression levels of type 3 AR transcript were expressed as a percentage relative to the expression level (set as a value of 100%) of wild-type AR (type 1 AR) transcript.

Further primers as shown in Figure 1A were designed to amplify the junction region of exon 1/exon 1b (E1E1b, forward primer 5′-AGGAAAGCGACTTCACCGCA-3′ and reverse primer 5′-TACAAAGTCCGGTACAAAGC-3′), exon 1b/exon 2 (E1bE2, forward primer 5′-GCTTTGTACCGGACTTTGTA-3′ and reverse primer 5′-AAGAAGACCTTGCAGCTTCC-3′), or exon 1/exon 1b/exon 2 (E1E2/E1E1bE2, forward primer 5′-TATGCTACTCCGGACCTTAC-3′ and reverse primer 5′-GTGGAAAGTAATAGTCAATGG-3′).

To quantify the mRNA levels of type 3a AR target genes in stably transfected LNCaP cells, gene specific primers were generated and qRT-PCR was performed as described above except that relative quantitation was performed using the 2^−ΔΔ*C*t^ method [82] with 18S rRNA as the reference housekeeping gene.

### 4.5. Preparation of pcDNA3 Vectors Expressing Type 1 AR or V7 Variant AR

The coding region of type 1 AR (wild-type AR) and AR-V7 were respectively amplified from the pCMV/AR3.1 vector containing the full-length wild-type AR and from a lentiviral expression vector containing the full-length AR-V7 (a kind gift of Yun Qiu, University of Maryland School of Medicine, Baltimore, MD, USA) using a common forward primer 5′-CTTAGGATCC*ATG*GAAGTGCAGTTAGGGCT-3′ paired with a reverse primer specific for AR-V7 (5′-ATATGCGGCCGC*TCA*GGGTCTGGTCATTTTGA-3′) or wild-type AR(5′-AAATGCGGCCGC*TCA*CTGGGTGTGGAAATAG-3′), and Phusion^®^ High-Fidelity DNA polymerase (Thermo Scientific). The initiation (*ATG*) and stop (*TCA*) codons of the wild-type *AR* or *AR-V7* in the forward and reverse primers respectively are in italics and BamHI and NotI sites (underlined) used for cloning in the forward and reverse primers respectively are underlined. The resultant PCR products were digested with BamHI and NotI and subsequently cloned into BamHI and NotI sites of pcDNA3 vector to generate two expression vectors: pcDNA3/AR-V7 and pcDNA3/type 1 AR. The identities of the constructs were verified by sequencing.

### 4.6. Preparation of pcDNA3 Vectors Expressing Type 3a AR or Type 3b AR

The In-Fusion HD Cloning Kit (Clontech) was used to assemble the coding sequence of type 3a AR in the pcDNA3 vector. The assembly approach was used because GC-rich repetitive sequences within exon 1 reduced the efficiency with which the full length AR sequence could be amplified and could lead to PCR-generated deletion artefacts. Furthermore, type 3 AR transcripts use a stop condon at nucleotides 34–36 of exon 2 precludes use of a reverse primer that is specific to type 3 AR. The coding sequence of exon 1 was amplified from pCMV/AR3.1 using the forward (5′-TACCGAGCTCGGATCC*ATG*GAAGTGCAGTTAGGGCTGGGAA-3′) and reverse (5′-GTCCGGAGTAGCTATCCATCCAGGGGCCCATTTCGCTTTTGA-3′) primers. The exon 1b/exon 2-encoded sequence of type 3a AR was amplified from VCaP cDNA using the forward (5′-TAGCTACTCCGGACCTTACGGGGACATGCGGTGCTGCGA-3′) and reverse (5′-CATGCTCGAGCGGCCGCTGGGGTGGAAAGTAATAG*TCA*ATGG-3′) primers. PCR was carried using Phusion^®^ High-Fidelity DNA polymerase (Thermo Scientific). In-Fusion HD Cloning Kits (Clontech) used a 10 µL ligation mixture that contained 2 µL of each of the two PCR amplicons, 1 µL of BamHI/NotI-digested pcDNA3 vector, 2 µL of 5× In-fusion HD enzyme premix, 3 µL of water. Ligation was carried out at 50 °C for 15 min. Positive clones were sequenced bidirectionally using primers T7 and Sp6 by the Flinders Sequencing Facility (Adelaide, SA, Australia). The pcDNA3/type 3b AR construct was then generated by insertion of TAG between exon 1 and exon 1b via site-directed mutagenesis using a complementary pair of primers (forward primer: 5′-ACCTTACGGGGACATGCGTAGGTGCTGCGAGCAGAGAGGGATT-3′, and reverse primer (5′-AATCCCTCTCTGCTCGCAGCACCTACGCATGTCCCCGTAAGGT-3′) (inserted TAG underlined).

### 4.7. Preparation of Androgen-Responsive Luciferase Reporters

The androgen-regulated enhancer of the *PSA* (prostate specific antigen) gene from nucleotides −3822 to −4301 relative to its initiation ATG codon [83] was amplified from genomic DNA (Roche, Pokolbin, NWS, Australia) using primers (forward, 5′-ATACACGCGTATCTTGTAGGGTGACCAGAGCA-3′/reverse, 5′-ATACTCGAGAGTATTTAATGGTGGAACGT-3′) and Phusion^®^ High-Fidelity DNA polymerase. Similarly, the androgen-regulated enhancer of the *TMPRSS2* (transmembrane protease, serine 2) gene from nucleotides −13,180 to −14,044 relative to its initiation ATG codon [84] was amplified using primers (forward, 5′-ATAGACGCGTCACTGATCTTATCTTGTAGGC-3′/reverse, 5′-ATAGCTCGAGCTTCTCCTCCCACCTCACTGCT-3′). The MluI and XhoI sites for cloning in the primers are underlined. The amplified *PSA* or *TMPRSS2* enhancer was subsequently cloned into the MluI and XhoI sites of pGL3-basic vector (Promega, Madison, WI, USA) generating two androgen-responsive luciferase reporters: pGL3/PSA enhancer and pGL3/TMPRSS2 enhancer.

### 4.8. Transient Transfection and Androgen-Responsive Reporter Luciferase Activity Assays

Cells (VCaP, LNCaP, MDA-MB-231) were plated into 96-well plates at 50%–60% confluence and incubated overnight in RPMI medium supplemented with 5% FBS. Cells of each well were then transfected with 100 ng of an androgen-responsive reporter (pGL3/PSA enhancer or pGL3/TMPRSS2 enhancer), 100 ng of a pcDNA3 vector (pcDNA3, pcDNA3/AR-V7, pcDNA3/type 3a AR, pcDNA3/type 3b AR) and 0.8 ng of pRL-null vector (Promega) as an internal reference, using Lipofectamine^®^2000 (Invitrogen). Twenty-four hours post-transfection, cells were cultured in phenol red-free RPMI 1640 medium with 5% dextran-coated charcoal (DCC)-stripped FBS (Sigma-Aldrich, Melbourne, VIC, Australia) for 48 h and then treated with either vehicle (0.1% ethanol) or 10 nM dihydrotestosterone (DHT) for 24 h. After treatment, cells were lysed in passive lysis buffer and assayed using the Dual-Luciferase Reporter Assay system (Promega). Firefly (*Photinus pyralis*) luciferase activity was first normalized using the Renilla (*Renilla reniformis*) activity and then expressed as-fold change over that of the cells transfected with the empty pcDNA3 vector and treated with ethanol.

### 4.9. Preparation of LNCaP Cells Stably Expressing Type 3 AR Protein and Monitoring of LNCaP Growth Using IncuCyte Imaging System

To make LNCaP cells that stably express type 3a or 3b AR and control LNCaP cells expressing no ectopic AR, pcDNA3/type 3a AR, pcDNA3/type 3b AR, or empty pcDNA3 plasmid was transfected into LNCaP cells and 48 h later, cells were treated with Geneticin (G418, 2 mg/mL). The pools of G418-resistant cells remaining 14 days post-transfection represented stably expressing type 3a AR, type 3b AR, or control cells (pools of cells transfected with empty pcDNA3 plasmid). The stable LNCaP cells were seeded in 96-well plates with 8 replicates for each type of cell at 4000 cells/well in RPMI-medium containing 5% stripped FBS and 10 nM DHT. Cells were cultured for 9 days and monitored using the IncuCyte^®^ imaging system (Essen BioScience, Brisbane, QLD, Australia) which scanned the wells every 2 h. Cell confluence as a factor of time was calculated using IncuCyte software (2013B).

### 4.10. Experimental Procedure of Microarrays and Data Analysis

LNCaP cells stably expressing type 3a AR (LNCaP/pcDNA3/type 3a AR cells) or no ectopic AR (LNCaP/pcDNA3 cells) were cultured in 6-well plates in phenol-red-free RPMI containing 5% stripped FBS for 72 h approaching 90% confluence, and then treated in triplicate with vehicle (0.1% ethanol) and 10 nM DHT for 24 h. After treatment, total RNA of treated cells was extracted using RNeasy Mini Kits (QIAGEN). Microarray experiments and data analysis were conducted by Adelaide University Microarray Facilities, Adelaide, SA, Australia), country. Briefly, 300 ng of total RNA was labelled using the Affymetrix WT Sense Target labeling assay as per the manufacturer’s instructions (Affymetrix Inc., Santa Clara, CA, USA). Samples were hybridised to Affymetrix Human Gene 2.0 ST Arrays for 17 h at 45 °C prior to washing, staining and scanning as per the manufacturer’s instructions. The array data was analysed using Partek Genomics Suite (Partek Inc., St. Louis, MO, USA). Briefly, cel files were imported using RMA back-ground correction, Partek’s own GC content correction, and mean probe summarization. Differential gene expression was assessed by ANOVA with the p value adjusted using step-up multiple test correction to control the false discovery rate (FDR) [85]. Adjusted *p* values <0.05 were considered to be significant. Complete lists of the 4 differentially expressed gene sets from the four different comparisons are given in Table S2, including genes whose expression was significantly affected by type 3a overexpression (relative to control vector) in the absence (set 1: C compared to A) or presence (set 2: D compared to B) of DHT and genes whose expression was regulated by DHT in the absence (set 3: B compared to A) or presence (set 4: D compared to C) of type 3a AR overexpression. The genes that were differentially regulated by type 3a AR overexpression in the absence (Set 1) and presence (Set 2) of DHT are defined as type 3a AR target genes and hence are listed in Table 1.

### 4.11. Western Blot Analysis of AR Variants

Cells were cultured in 6-well plates at 50%–60% confluence and incubated overnight in RPMI medium supplemented with 5% FBS. Cells were then transfected with 2 µg of relevant expression plasmids as indicated in the figures. After 48 h, whole cell lysates were prepared using RIPA buffer (25 mM Tris-HCl (pH 7.6), 150 mM NaCl, 1% NP-40, 1% sodium deoxycholate, and 0.1% SDS). Thirty micrograms of protein for each sample were resolved on 10% SDS-polyacrylamide gels and subjected to standard immunoblot analysis using a primary antibody recognizing the AR (N20, Santa Cruz Biotechnology, VIC, Australia) or β-actin (Sigma-Aldrich), and a secondary donkey anti-rabbit antibody conjugated with peroxidase (Neomarkers, Fremont, CA, USA). SuperSignalWest Pico Chemiluminescent substrate (Thermo Scientific) was used for immunodetection, and immuno-signals were photographed by an ImageQuant LAS 4000 luminescent image analyzer (GE Healthcare Life Sciences, Piscataway, NJ, USA). The anti-AR (N20) antibody raised against the N-terminus of the human AR is able to detect wild-type AR (type 1 AR) and three AR variants, AR-V7, type 3a AR, and type 3b AR.

### 4.12. Determination of Cellular Localization of Type 3 AR Proteins in PC3 Cells Using GFP-Tagged Vectors and Fluorescence Imaging

The In-Fusion HD Cloning Kit (Clontech) was used to prepare the pcDNA3 vectors that expressed fusion proteins with GFP fused at the N-termimus of wild-type AR (pcDNA3/GFP/AR WT), type 3a AR (pcDNA3/GFP/type 3a AR), or type 3b AR (pcDNA3/GFP/type 3b AR). The full coding sequence of GFP without the stop codon was amplified from pEGFP/AR-V7 using the forward (5′-TACCGAGCTCGGATCC*ATG*GTGAGCAAGGGCGAGG-3′) and reverse (5′-CGTTACTAGTCCTAGGCTTGTACAGCTCGTCCATGCCGAGA-3′) primers. PCR was carried out using Phusion^®^ High-Fidelity DNA polymerase (Thermo Scientific). pcDNA3/type 3a AR, pcDNA3/type 3b AR, or pcDNA3/type 1 AR (AR WT) was digested by BamHI and the digested products were purified. The amplified GFP PCR products were ligated with BamHI-digested pcDNA3 vector using In-Fusion HD Cloning Kits as described above. Expression consutructs were verified by bidirectional sequencing. Glass coverslips were coated with 50 µg/mL rat-tail collagen for 1 h at 37 °C and seeded with PC3 cells in 24-well plates in RPMI media containing 10% FBS. PC3 cells were transfected in duplicate wells using 1.6 µL Lipofectamine 2000 (Invitrogen) and 400 ng of GFP-tagged vector (pcDNA3/GFP/type 3a AR, pcDNA3/GFP/type 3b AR, or pcDNA3/GFP/wild-type AR) alone or in combination (untagged pcDNA3/wild-type AR with pcDNA3/GFP/type 3a AR, or pcDNA3/GFP/type 3b AR). Untagged pcDNA3 empty vector was added where necessary to normalize total DNA amounts between conditions. At 24 h post-transfection media was replaced with phenol red-free RPMI containing 10% stripped FBS, and at 48 h post-transfection cells were treated with 10 nM DHT or vehicle for 4 h. After treatment, cells were fixed with 3.7% formaldehyde in PBS for 10 min at room temperature and nuclei were counterstained with 1 µg/mL DAPI for 5 min before mounting. Cells were imaged with an Olympus BX-50 upright microscope and the mean fluorescence intensity of nuclear and cytoplasmic GFP of each cell was quantified using ImageJ (NIH). Background fluorescence was subtracted and results were reported as means ± SEM of ratios of nuclear/cytoplasmic fluorescence from 20 to 35 cells for each experimental condition.

## 5. Conclusions

In summary, we have identified novel (type 3) variant AR proteins that can act as androgen-independent transcriptional activators, in common with other LBD-lacking AR variants. Type 3 AR variants also lack the DBD, but may interact with wildtype AR or other AR variants in order to interact with DNA and modulate gene expression. The mechanism by which these novel variants inhibit androgen-induced LNCaP cell growth, and in particular the roles of identified type 3 AR target genes, will require further study. Similar to other LDB-lacking AR variants, type 3a AR transcripts are expressed at a level of <5% of the wild-type AR in normal breast and prostate tissues and in the cancer cell lines studied here. However, potential upregulation/downregulation of type 3 AR in clinical breast and prostate cancer specimens remains to be investigated.

## Figures and Tables

**Table 1:** Genes and microRNAs regulated by type 3a AR in LNCaP cells as revealed by microarray analysis using the Affymetrix Gene Array 2.0.

Gene Symbol	RefSeqgene	Full Name	C/A Comparison		D/B Comparison	
			Fold Change	*p* Value	Fold Change	*p* Value
*PCGEM1*	NR_002769	Prostate cancer gene expression marker 1	−2.21	0.00039	−2.61	0.00009
*BCHE*	NM_000055	Butyrylcholinesterase	−2.86	0.00039	−3.61	0.00009
*EFNB2*	NM_004093	Ephrin-B2	−4.32	0.00163	−4.77	0.00094
*PEG3*	NM_006210	Paternally expressed 3, transcript 1	2.21	0.00452	2.06	0.00838
*PSMD5*	NM_005047	Proteasome (prosome, macropain) 26S subunit, non-AT	−2.61	0.00163	−2.68	0.00134
*MBNL2*	NM_144778	Muscleblind-like 2 (Drosophila) (MBNL2), transcript	−2.22	0.00452	−2.43	0.00399
*GSTA1*	NM_145740	Glutathione *S*-transferase α 1	−2.21	0.00631	−2.40	0.00572
*TLL1*	NM_012464	Tolloid-like 1	−3.13	0.00518	−2.77	0.01127
*ZNF608*	NM_020747	Zinc finger protein 608	−2.21	0.02334	−2.27	0.02299
		Has-miR-3689b	−4.23	0.02106	−4.14	0.02301
		Has-miR-3689a	−3.58	0.02117	−3.21	0.03057
*SYT4*	NM_020783	Synaptotagmin IV	−2.39	0.00249		
*ST8SIA4*	NM_005668	ST8 α-*N*-acetyl-neuraminide α-2.8-sialytransferase	−6.35	0.00318		
*LRRTM3*	NM_178011	Leucine rich repeat transmembrane neuronal 3	−2.74	0.00452		
*EPHA3*	NM_005233	EPH receptor A3, transcript variant 1			2.1	0.00012
*COL12A1*	NM_004370	Collagen, type XII, α 1			−2.27	0.01362
*GRIN3A*	NM_133445	Glutamate receptor, ionotropic, *N*-methyl-*D*-aspartat			−2.01	0.01362
*SLITRK3*	NM_014926	SLIT and NTRK-like family, member 3			−2.06	0.00094
*FAM198B*	NM_001031700	Family with sequence similarity 198, member B			−2.09	0.01362
*LRRN1*	NM_020873	Leucine rich repeat neuronal 1			−2.04	0.02305
*TOX3*	NM_001146188	TOX high mobility group box family member 3			−2.09	0.03989

A: LNCaP/pcDNA3 cells in the absence of DHT; B: LNCaP/pcDNA3 cells in the presence of DHT; C: LNCaP/pcDNA3/type 3a AR cells in the absence of DHT; D: LNCaP/pcDNA3/type 3a AR cells in the presence of DHT.

## Supplementary Materials

Supplementary materials can be found at www.mdpi.com/1422-0067/18/1/40/s1.

## References

[1] Kaarbo M., Klokk T.I., Saatcioglu F. (2007). Androgen signaling and its interactions with other signaling pathways in prostate cancer. BioEssays News Rev. Mol. Cell. Dev. Biol..

[2] Katzenwadel A., Wolf P. (2015). Androgen deprivation of prostate cancer: Leading to a therapeutic dead end. Cancer Lett..

[3] Gunter J.H., Lubik A.A., McKenzie I., Pollak M., Nelson C.C. (2012). The Interactions between insulin and androgens in progression to castrate-resistant prostate cancer. Adv. Urol..

[4] Chandrasekar T., Yang J.C., Gao A.C., Evans C.P. (2015). Mechanisms of resistance in castration-resistant prostate cancer (CRPC). Transl. Androl. Urol..

[5] Hu R., Isaacs W.B., Luo J. (2011). A snapshot of the expression signature of androgen receptor splicing variants and their distinctive transcriptional activities. Prostate.

[6] Lu J., Van der Steen T., Tindall D.J. (2015). Are androgen receptor variants a substitute for the full-length receptor?. Nat. Rev. Urol..

[7] Dehm S.M., Tindall D.J. (2011). Alternatively spliced androgen receptor variants. Endocr. Relat. Cancer.

[8] Ahrens-Fath I., Politz O., Geserick C., Haendler B. (2005). Androgen receptor function is modulated by the tissue-specific AR45 variant. FEBS J..

[9] Hu D.G., Hickey T.E., Irvine C., Wijayakumara D.D., Lu L., Tilley W.D., Selth L.A., Mackenzie P.I. (2014). Identification of androgen receptor splice variant transcripts in breast cancer cell lines and human tissues. Horm. Cancer.

[10] Lu C., Luo J. (2013). Decoding the androgen receptor splice variants. Transl. Androl. Urol..

[11] Li Y., Hwang T.H., Oseth L.A., Hauge A., Vessella R.L., Schmechel S.C., Hirsch B., Beckman K.B., Silverstein K.A., Dehm S.M. (2012). AR intragenic deletions linked to androgen receptor splice variant expression and activity in models of prostate cancer progression. Oncogene.

[12] Liu L.L., Xie N., Sun S., Plymate S., Mostaghel E., Dong X. (2014). Mechanisms of the androgen receptor splicing in prostate cancer cells. Oncogene.

[13] Nyquist M.D., Li Y., Hwang T.H., Manlove L.S., Vessella R.L., Silverstein K.A., Voytas D.F., Dehm S.M. (2013). TALEN-engineered *AR* gene rearrangements reveal endocrine uncoupling of androgen receptor in prostate cancer. Proc. Natl. Acad. Sci. USA.

[14] Dehm S.M., Schmidt L.J., Heemers H.V., Vessella R.L., Tindall D.J. (2008). Splicing of a novel androgen receptor exon generates a constitutively active androgen receptor that mediates prostate cancer therapy resistance. Cancer Res..

[15] Guo Z., Yang X., Sun F., Jiang R., Linn D.E., Chen H., Chen H., Kong X., Melamed J., Tepper C.G. (2009). A novel androgen receptor splice variant is up-regulated during prostate cancer progression and promotes androgen depletion-resistant growth. Cancer Res..

[16] Sun S., Sprenger C.C., Vessella R.L., Haugk K., Soriano K., Mostaghel E.A., Page S.T., Coleman I.M., Nguyen H.M., Sun H. (2010). Castration resistance in human prostate cancer is conferred by a frequently occurring androgen receptor splice variant. J. Clin. Investig..

[17] Doane A.S., Danso M., Lal P., Donaton M., Zhang L., Hudis C., Gerald W.L. (2006). An estrogen receptor-negative breast cancer subset characterized by a hormonally regulated transcriptional program and response to androgen. Oncogene.

[18] Farmer P., Bonnefoi H., Becette V., Tubiana-Hulin M., Fumoleau P., Larsimont D., Macgrogan G., Bergh J., Cameron D., Goldstein D. (2005). Identification of molecular apocrine breast tumours by microarray analysis. Oncogene.

[19] Hickey T.E., Irvine C.M., Dvinge H., Tarulli G.A., Hanson A.R., Ryan N.K., Pickering M.A., Birrell S.N., Hu D.G., Mackenzie P.I. (2015). Expression of androgen receptor splice variants in clinical breast cancers. Oncotarget.

[20] Weiss B., Faus H., Haendler B. (2007). Phylogenetic conservation of the androgen receptor AR45 variant form in placental mammals. Gene.

[21] Watson P.A., Chen Y.F., Balbas M.D., Wongvipat J., Socci N.D., Viale A., Kim K., Sawyers C.L. (2010). Constitutively active androgen receptor splice variants expressed in castration-resistant prostate cancer require full-length androgen receptor. Proc. Natl. Acad. Sci. USA.

[22] Isken O., Maquat L.E. (2008). The multiple lives of NMD factors: Balancing roles in gene and genome regulation. Nat. Rev. Genet..

[23] Subik K., Lee J.F., Baxter L., Strzepek T., Costello D., Crowley P., Xing L., Hung M.C., Bonfiglio T., Hicks D.G. (2010). The expression patterns of ER, PR, HER2, CK5/6, EGFR, Ki-67 and AR by immunohistochemical analysis in breast cancer cell lines. Breast Cancer Basic Clin. Res..

[24] Srikantan V., Zou Z., Petrovics G., Xu L., Augustus M., Davis L., Livezey J.R., Connell T., Sesterhenn I.A., Yoshino K. (2000). PCGEM1, a prostate-specific gene, is overexpressed in prostate cancer. Proc. Natl. Acad. Sci. USA.

[25] Parolia A., Crea F., Xue H., Wang Y., Mo F., Ramnarine V.R., Liu H.H., Lin D., Saidy N.R., Clermont P.L. (2015). The long non-coding RNA PCGEM1 is regulated by androgen receptor activity in vivo. Mol. Cancer.

[26] Wu L., Runkle C., Jin H.J., Yu J., Li J., Yang X., Kuzel T., Lee C., Yu J. (2014). CCN3/NOV gene expression in human prostate cancer is directly suppressed by the androgen receptor. Oncogene.

[27] Ulrix W., Swinnen J.V., Heyns W., Verhoeven G. (1999). Androgens down-regulate the expression of the human homologue of paternally expressed gene-3 in the prostatic adenocarcinoma cell line LNCaP. Mol. Cell. Endocrinol..

[28] Kosugi S., Hasebe M., Matsumura N., Takashima H., Miyamoto-Sato E., Tomita M., Yanagawa H. (2009). Six classes of nuclear localization signals specific to different binding grooves of importin α. J. Biol. Chem..

[29] Wente S.R., Rout M.P. (2010). The nuclear pore complex and nuclear transport. Cold Spring Harb. Perspect. Biol..

[30] Zhou Z.X., Sar M., Simental J.A., Lane M.V., Wilson E.M. (1994). A ligand-dependent bipartite nuclear targeting signal in the human androgen receptor. Requirement for the DNA-binding domain and modulation by NH_2_-terminal and carboxyl-terminal sequences. J. Biol. Chem..

[31] Hu R., Dunn T.A., Wei S., Isharwal S., Veltri R.W., Humphreys E., Han M., Partin A.W., Vessella R.L., Isaacs W.B. (2009). Ligand-independent androgen receptor variants derived from splicing of cryptic exons signify hormone-refractory prostate cancer. Cancer Res..

[32] Korlimarla A., Bhandary L., Prabhu J.S., Shankar H., Sankaranarayanan H., Kumar P., Remacle J., Natarajan D., Sridhar T.S. (2013). Identification of a non-canonical nuclear localization signal (NLS) in BRCA1 that could mediate nuclear localization of splice variants lacking the classical NLS. Cell. Mol. Biol. Lett..

[33] Xu D., Zhan Y., Qi Y., Cao B., Bai S., Xu W., Gambhir S.S., Lee P., Sartor O., Flemington E.K. (2015). Androgen receptor splice variants dimerize to transactivate target genes. Cancer Res..

[34] Heisler L.E., Evangelou A., Lew A.M., Trachtenberg J., Elsholtz H.P., Brown T.J. (1997). Androgen-dependent cell cycle arrest and apoptotic death in PC-3 prostatic cell cultures expressing a full-length human androgen receptor. Mol. Cell. Endocrinol..

[35] Yuan S., Trachtenberg J., Mills G.B., Brown T.J., Xu F., Keating A. (1993). Androgen-induced inhibition of cell proliferation in an androgen-insensitive prostate cancer cell line (PC-3) transfected with a human androgen receptor complementary DNA. Cancer Res..

[36] Urbanucci A., Sahu B., Seppala J., Larjo A., Latonen L.M., Waltering K.K., Tammela T.L., Vessella R.L., Lahdesmaki H., Janne O.A. (2012). Overexpression of androgen receptor enhances the binding of the receptor to the chromatin in prostate cancer. Oncogene.

[37] Waltering K.K., Helenius M.A., Sahu B., Manni V., Linja M.J., Janne O.A., Visakorpi T. (2009). Increased expression of androgen receptor sensitizes prostate cancer cells to low levels of androgens. Cancer Res..

[38] Chan S.C., Li Y., Dehm S.M. (2012). Androgen receptor splice variants activate androgen receptor target genes and support aberrant prostate cancer cell growth independent of canonical androgen receptor nuclear localization signal. J. Biol. Chem..

[39] Bianchi-Frias D., Coleman I., Banerji J., Pham K., Jeldres C., Gulati R., Xia J., Tomlins J., Porter C., Nelson P. (2015). Novel urine markers for diagnosing and monitoring non-indolent prostate cancer. J. Clin. Oncol..

[40] Koie T., Ohyama C., Hatakeyama S., Imai A., Yoneyama T., Hashimoto Y., Yoneyama T., Tobisawa Y., Hosogoe S., Yamamoto H. (2016). Significance of preoperative butyrylcholinesterase as an independent predictor of biochemical recurrence-free survival in patients with prostate cancer treated with radical prostatectomy. Int. J. Clin. Oncol..

[41] Takahara K., Azuma H., Sakamoto T., Kiyama S., Inamoto T., Ibuki N., Nishida T., Nomi H., Ubai T., Segawa N. (2009). Conversion of prostate cancer from hormone independency to dependency due to AMACR inhibition: Involvement of increased AR expression and decreased IGF1 expression. Anticancer Res..

[42] Parsons J.K., Nelson C.P., Gage W.R., Nelson W.G., Kensler T.W., de Marzo A.M. (2001). GSTA1 expression in normal, preneoplastic, and neoplastic human prostate tissue. Prostate.

[43] Cheville J.C., Karnes R.J., Therneau T.M., Kosari F., Munz J.M., Tillmans L., Basal E., Rangel L.J., Bergstralh E., Kovtun I.V. (2008). Gene panel model predictive of outcome in men at high-risk of systemic progression and death from prostate cancer after radical retropubic prostatectomy. J. Clin. Oncol. Off. J. Am. Soc. Clin. Oncol..

[44] Kosari F., Munz J.M., Savci-Heijink C.D., Spiro C., Klee E.W., Kube D.M., Tillmans L., Slezak J., Karnes R.J., Cheville J.C. (2008). Identification of prognostic biomarkers for prostate cancer. Clin. Cancer Res. Off. J. Am. Assoc. Cancer Res..

[45] Xu A., Sun S. (2015). Genomic profiling screens small molecules of metastatic prostate carcinoma. Oncol. Lett..

[46] Rutkowski R., Mertens-Walker I., Lisle J.E., Herington A.C., Stephenson S.A. (2012). Evidence for a dual function of EphB4 as tumor promoter and suppressor regulated by the absence or presence of the ephrin-B2 ligand. Int. J. Cancer.

[47] Dozmorov M.G., Hurst R.E., Culkin D.J., Kropp B.P., Frank M.B., Osban J., Penning T.M., Lin H.K. (2009). Unique patterns of molecular profiling between human prostate cancer LNCaP and PC-3 cells. Prostate.

[48] Wu R., Wang H., Wang J., Wang P., Huang F., Xie B., Zhao Y., Li S., Zhou J. (2014). EphA3, induced by PC-1/PrLZ, contributes to the malignant progression of prostate cancer. Oncol. Rep..

[49] Bawa P., Zackaria S., Verma M., Gupta S., Srivatsan R., Chaudhary B., Srinivasan S. (2015). Integrative analysis of normal long intergenic non-coding RNAs in prostate cancer. PLoS ONE.

[50] Sharad S., Srivastava A., Ravulapalli S., Parker P., Chen Y., Li H., Petrovics G., Dobi A. (2011). Prostate cancer gene expression signature of patients with high body mass index. Prostate Cancer Prostatic Dis..

[51] Petrovics G., Zhang W., Makarem M., Street J.P., Connelly R., Sun L., Sesterhenn I.A., Srikantan V., Moul J.W., Srivastava S. (2004). Elevated expression of PCGEM1, a prostate-specific gene with cell growth-promoting function, is associated with high-risk prostate cancer patients. Oncogene.

[52] Xue Y., Wang M., Kang M., Wang Q., Wu B., Chu H., Zhong D., Qin C., Yin C., Zhang Z. (2013). Association between lncrna PCGEM1 polymorphisms and prostate cancer risk. Prostate Cancer Prostatic Dis..

[53] Fu X., Ravindranath L., Tran N., Petrovics G., Srivastava S. (2006). Regulation of apoptosis by a prostate-specific and prostate cancer-associated noncoding gene, PCGEM1. DNA Cell Biol..

[54] He J.H., Zhang J.Z., Han Z.P., Wang L., Lv Y.B., Li Y.G. (2014). Reciprocal regulation of PCGEM1 and miR-145 promote proliferation of LNCaP prostate cancer cells. J. Exp. Clin. Cancer Res. CR.

[55] Yang L., Lin C., Jin C., Yang J.C., Tanasa B., Li W., Merkurjev D., Ohgi K.A., Meng D., Zhang J. (2013). lncRNA-dependent mechanisms of androgen-receptor-regulated gene activation programs. Nature.

[56] Zhang Z., Zhou N., Huang J., Ho T.T., Zhu Z., Qiu Z., Zhou X., Bai C., Wu F., Xu M. (2016). Regulation of androgen receptor splice variant AR3 by PCGEM1. Oncotarget.

[57] Relaix F., Wei X., Li W., Pan J., Lin Y., Bowtell D.D., Sassoon D.A., Wu X. (2000). Pw1/Peg3 is a potential cell death mediator and cooperates with Siah1a in p53-mediated apoptosis. Proc. Natl. Acad. Sci. USA.

[58] Relaix F., Wei X.J., Wu X., Sassoon D.A. (1998). Peg3/Pw1 is an imprinted gene involved in the TNF-NFκB signal transduction pathway. Nat. Genet..

[59] Forse G.J., Uson M.L., Nasertorabi F., Kolatkar A., Lamberto I., Pasquale E.B., Kuhn P. (2015). Distinctive structure of the EphA3/Ephrin-A5 complex reveals a dual mode of Eph receptor interaction for ephrin-A5. PLoS ONE.

[60] Tomlins S.A., Mehra R., Rhodes D.R., Cao X., Wang L., Dhanasekaran S.M., Kalyana-Sundaram S., Wei J.T., Rubin M.A., Pienta K.J. (2007). Integrative molecular concept modeling of prostate cancer progression. Nat. Genet..

[61] Krusche B., Ottone C., Clements M.P., Johnstone E.R., Goetsch K., Lieven H., Mota S.G., Singh P., Khadayate S., Ashraf A. (2016). EphrinB2 drives perivascular invasion and proliferation of glioblastoma stem-like cells. eLife.

[62] Battisti V., Bagatini M.D., Maders L.D., Chiesa J., Santos K.F., Goncalves J.F., Abdalla F.H., Battisti I.E., Schetinger M.R., Morsch V.M. (2012). Cholinesterase activities and biochemical determinations in patients with prostate cancer: Influence of Gleason score, treatment and bone metastasis. Biomed. Pharmacother..

[63] Chen Q., Watson J.T., Marengo S.R., Decker K.S., Coleman I., Nelson P.S., Sikes R.A. (2006). Gene expression in the LNCaP human prostate cancer progression model: Progression associated expression in vitro corresponds to expression changes associated with prostate cancer progression in vivo. Cancer Lett..

[64] Di Pietro G., Magno L.A., Rios-Santos F. (2010). Glutathione *S*-transferases: An overview in cancer research. Expert Opin. Drug Metab. Toxicol..

[65] Deng Q., He B., Pan Y., Sun H., Liu X., Chen J., Ying H., Lin K., Peng H., Wang S. (2015). Polymorphisms of GSTA1 contribute to elevated cancer risk: Evidence from 15 studies. J. B.U.ON. Off. J. Balk. Union Oncol..

[66] Komiya Y., Tsukino H., Nakao H., Kuroda Y., Imai H., Katoh T. (2005). Human glutathione *S*-transferase A1, T1, M1, and P1 polymorphisms and susceptibility to prostate cancer in the Japanese population. J. Cancer Res. Clin. Oncol..

[67] Sa R.A., Moreira Ados S., Cabello P.H., Ornellas A.A., Costa E.B., Matos Cda S., Alves G., Hatagima A. (2014). Human glutathione *S*-transferase polymorphisms associated with prostate cancer in the Brazilian population. Int. Braz. J. Urol. Off. J. Braz. Soc. Urol..

[68] Aruga J., Yokota N., Mikoshiba K. (2003). Human SLITRK family genes: Genomic organization and expression profiling in normal brain and brain tumor tissue. Gene.

[69] Milde T., Shmelkov S.V., Jensen K.K., Zlotchenko G., Petit I., Rafii S. (2007). A novel family of slitrk genes is expressed on hematopoietic stem cells and leukemias. Leukemia.

[70] Wang C.J., Zhang Z.Z., Xu J., Wang M., Zhao W.Y., Tu L., Zhuang C., Liu Q., Shen Y.Y., Cao H. (2015). SLITRK3 expression correlation to gastrointestinal stromal tumor risk rating and prognosis. World J. Gastroenterol..

[71] Schreiber S.C., Giehl K., Kastilan C., Hasel C., Muhlenhoff M., Adler G., Wedlich D., Menke A. (2008). Polysialylated NCAM represses *E*-cadherin-mediated cell–cell adhesion in pancreatic tumor cells. Gastroenterology.

[72] Carter H., Chen S., Isik L., Tyekucheva S., Velculescu V.E., Kinzler K.W., Vogelstein B., Karchin R. (2009). Cancer-specific high-throughput annotation of somatic mutations: Computational prediction of driver missense mutations. Cancer Res..

[73] Yu X., Li Z. (2015). *TOX* gene: A novel target for human cancer gene therapy. Am. J. Cancer Res..

[74] Bayraktar S., Thompson P.A., Yoo S.Y., Do K.A., Sahin A.A., Arun B.K., Bondy M.L., Brewster A.M. (2013). The relationship between eight GWAS-identified single-nucleotide polymorphisms and primary breast cancer outcomes. Oncologist.

[75] Chen M.B., Wu X.Y., Shen W., Wei M.X., Li C., Cai B., Tao G.Q., Lu P.H. (2011). Association between polymorphisms of trinucleotide repeat containing 9 gene and breast cancer risk: Evidence from 62,005 subjects. Breast Cancer Res. Treat..

[76] Easton D.F., Pooley K.A., Dunning A.M., Pharoah P.D., Thompson D., Ballinger D.G., Struewing J.P., Morrison J., Field H., Luben R. (2007). Genome-wide association study identifies novel breast cancer susceptibility loci. Nature.

[77] Elematore I., Gonzalez-Hormazabal P., Reyes J.M., Blanco R., Bravo T., Peralta O., Gomez F., Waugh E., Margarit S., Ibanez G. (2014). Association of genetic variants at TOX3, 2q35 and 8q24 with the risk of familial and early-onset breast cancer in a South-American population. Mol. Biol. Rep..

[78] He X., Yao G., Li F., Li M., Yang X. (2014). Risk-association of five SNPs in TOX3/LOC643714 with breast cancer in southern China. Int. J. Mol. Sci..

[79] Reeves G.K., Travis R.C., Green J., Bull D., Tipper S., Baker K., Beral V., Peto R., Bell J., Zelenika D. (2010). Incidence of breast cancer and its subtypes in relation to individual and multiple low-penetrance genetic susceptibility loci. JAMA.

[80] Gudmundsdottir E.T., Barkardottir R.B., Arason A., Gunnarsson H., Amundadottir L.T., Agnarsson B.A., Johannsson O.T., Reynisdottir I. (2012). The risk allele of SNP rs3803662 and the mRNA level of its closest genes TOX3 and LOC643714 predict adverse outcome for breast cancer patients. BMC Cancer.

[81] Hu D.G., Mackenzie P.I. (2009). Estrogen receptor α, fos-related antigen-2, and c-Jun coordinately regulate human UDP glucuronosyltransferase 2B15 and 2B17 expression in response to 17β-estradiol in MCF-7 cells. Mol. Pharmacol..

[82] Livak K.J., Schmittgen T.D. (2001). Analysis of relative gene expression data using real-time quantitative PCR and the 2^−ΔΔ*C*t^ Method. Methods.

[83] Cleutjens K.B., van der Korput H.A., van Eekelen C.C., van Rooij H.C., Faber P.W., Trapman J. (1997). An androgen response element in a far upstream enhancer region is essential for high, androgen-regulated activity of the prostate-specific antigen promoter. Mol. Endocrinol..

[84] Wang Q., Li W., Liu X.S., Carroll J.S., Janne O.A., Keeton E.K., Chinnaiyan A.M., Pienta K.J., Brown M. (2007). A hierarchical network of transcription factors governs androgen receptor-dependent prostate cancer growth. Mol. Cell.

[85] Benjamini Y., Hochberg Y. (1995). Controlling the false discovery rate: A practical and powerful approach to multiple testing. J. R. Stat. Soc. Ser. B.

