# Supplementary material for: Novel Nine-Exon AR Transcripts (Exon 1/Exon 1b/Exons 2–8) in Normal and Cancerous Breast and Prostate Cells
Source: Int J Mol Sci. 2016 Dec 27;18(1):40. doi: 10.3390/ijms18010040 (PMC5297675; doi:10.3390/ijms18010040)
Supplement: Supplementary file 1 [file ijms-18-00040-s001.zip › ijms-154219-Supplementary Materials/ijms-154219-supplementary.pdf]

Supplementary Materials: Novel Nine-Exon AR Transcripts (Exon 1/Exon 1b/Exons 2–8) in Normal and Cancerous Breast and Prostate Cells

Dong Gui Hu, Ross A. McKinnon, Julie-Ann Hulin, Peter I. Mackenzie and Robyn Meech

supplemental Figure 1

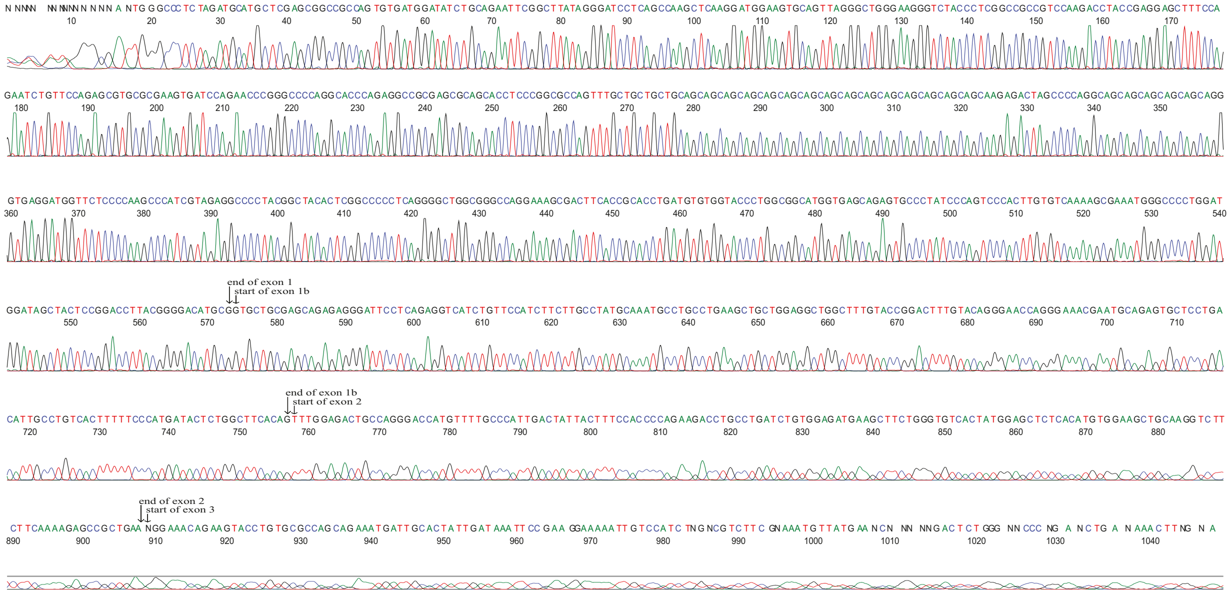

Figure Supplementary 1: Chromatogram of sequencing shows splicing of exon 1b of the AR gene between exon 1 and exon 2 in a clone that was derived from breast cancer MDA-MB-453 cells in RT-PCR designed to amplify the full-length cDNA of the AR gene

Figure S1. Chromatogram of sequencing shows splicing of exon 1b of the *AR* gene between exon 1 and exon 2 in a clone that was derived from breast cancer MDA-MB-453 cells in RT-PCR designed to amplify the full-length cDNA of the *AR* gene. The nucleotides of A, C, G, T in the chromatogram are in green, blue, black, and red, respectively.

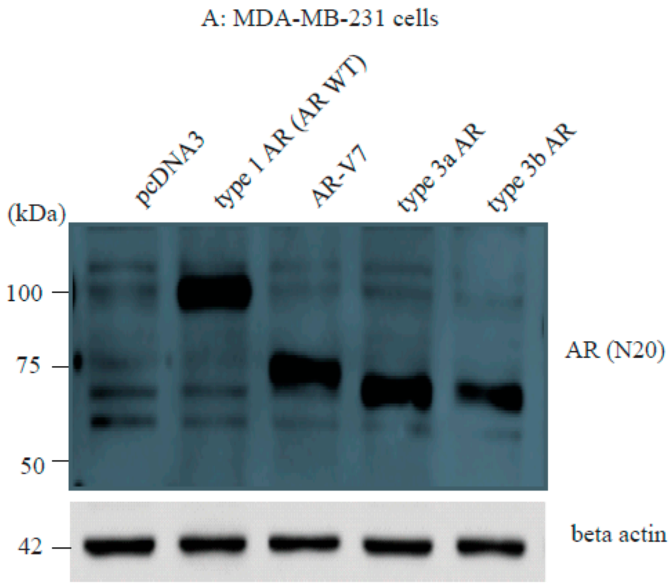

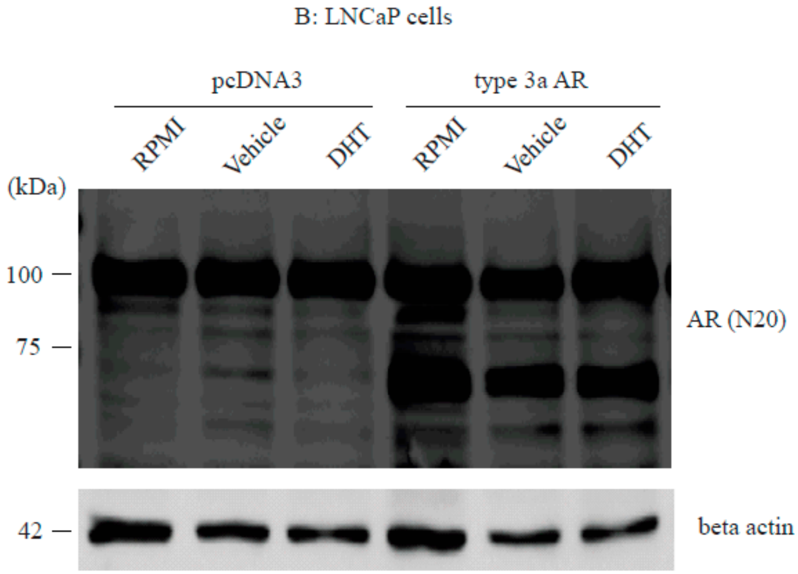

Figure S2. The anti-AR antibody (N20) detected low levels of proteins with a molecular weight consistent with type 3 AR proteins in MDA-MB-231 cells (**A**) and LNCaP cells (**B**) in western blotting assays with a 10-minute exposure time. (**A**) MDA-MB-231 cells were transfected with a pcDNA3 vector expressing no ectopic protein (pcDNA3), wild-type AR (AR WT), AR-V7, type 3a AR, or type 3b AR; (**B**) LNCaP cells were transfected with a pcDNA3 vector expressing no ectopic protein (pcDNA3) or type 3a AR. Cell lysates were prepared from transfected cells 48 hours post-transfection and 30 μg of proteins were subjected to Western blotting assays with an antibody recognizing androgen receptor (AR) (N20) or beta actin as described in Methods. Of note, a 10-minute exposure time was applied in order to show low levels of endogenously expressed type 3 AR proteins in MDA-MB-231 (**A**) and LNCaP (**B**) cells.

Table S1. Primers used for real-time quantitative PCR.

Genes	Forward Primer (5′–3′)	Reverse Primer (5′–3′)	PCR Products Size (bp)
BCHE	ACGGTGGGCAAATTTTGCAA	CATTGTTGAGCACGTAGTTT	149
COL12A1	CAGAATAACGGTGGACCCTA	GTTATTGTCACCACATACTC	120
EFNB2	ACAAATGGAAGAAGTTCGAC	GAAGATGATGCATCCTGAAG	150
EPHA3	AGGTGTGCAATGTCATGGAC	ACCAATGGAATGCTATTGCA	130
GRIN3A	TGGTGAGCACATAGTATACA	ACACGTTTGGTCTTGAAATG	150
GSTA1	ACCTCTATGGGAAAGACATA	TGGCATCTTTTTCCTCAGGT	120
LRRTM3	CCCTAAGCCAAAGCAAAAGA	AGCATTGTCAGTAAGACAGT	130
LRRN1	TGCCACGGACCTGTGCACAT	GTCTTGGTTGAGCTGTGTAG	130
PSMD5	TCACCCTGATGATTCTGTA	ATAGATTCTCTCCACCAATG	140
PCG-AS	GCCAATCCGTATATTGTAAA	TGTAGGCATGATAGATAACA	140
PCG3	TGGTCTGAAGTTCCAGAAAG	GCTTTGGAGGCAGCATTTCT	140
PCGEM1	CCGTAACCTGTGTCTGCAACTTCCT	CATGAAGTGTCAAATGCACCAAGCC	145
SLITRK3	CCTGGCGCTCCAGTTTAGGA	ATCCTAGAGCAATTGTGCTT	130
ST8SIA4	TTCAATCTTCCAGCACAATG	AGGCTTAAAACTGCTCTTGA	130
TOX3	AGACTTCATTACCTGATAAC	ATTTCGAATTCCTCGTCCCC	140
